# Temporal multi-omics analysis of COVID-19 in end-stage kidney disease

**DOI:** 10.1016/j.xgen.2025.100918

**Published:** 2025-06-17

**Authors:** Emily Stephenson, Erin Macdonald-Dunlop, Lisa M. Dratva, Rik G.H. Lindeboom, Zewen Kelvin Tuong, Win Min Tun, Lorenz Kretschmer, Norzawani B. Buang, Stephane Ballereau, Mia Cabantaus, Ana Peñalver, Elena Prigmore, John R. Ferdinand, Benjamin J. Stewart, Jack Gisby, Talat H. Malik, Candice L. Clarke, Nicholas Medjeral-Thomas, Maria Prendecki, Stephen McAdoo, Anais Portet, Michelle Willicombe, Eleanor Sandhu, Matthew C. Pickering, Marina Botto, Sarah A. Teichmann, Muzlifah Haniffa, Menna R. Clatworthy, David C. Thomas, James E. Peters

**Affiliations:** 1 Biosciences Institute, Newcastle University, Newcastle upon Tyne, UK; 2 Cellular Genetics, Wellcome Sanger Institute, Hinxton, UK; 3 Department of Immunology and Inflammation, Imperial College London, London, UK; 4 Department of Medicine, University of Cambridge, Cambridge, UK; 5 Cambridge Stem Cell Institute, University of Cambridge, Cambridge, UK; 6 Cambridge Institute of Therapeutic Immunology and Infectious Disease, University of Cambridge, Cambridge, UK; 7 Imperial College Renal and Transplant Centre, Imperial College Healthcare National Health Service (NHS) Trust, Hammersmith Hospital, London, UK; 8 Department of Dermatology and National Institute for Health and Care Research (NIHR) Newcastle Biomedical Research Centre, Newcastle Hospitals NHS Foundation Trust, Newcastle upon Tyne, UK; 9 CIFAR Macmillan Multiscale Human Programme, CIFAR, Toronto, Canada

**Keywords:** COVID-19, SARS-CoV-2, end-stage kidney disease, ESKD, single-cell transcriptomics, T cell receptor, TCR, sequencing, CITE-seq, longitudinal, monocytes, glucocorticoids, interferon

## Abstract

Patients with end-stage kidney disease (ESKD) are at high risk of severe COVID-19. We performed longitudinal single-cell immune profiling of ESKD patients with COVID-19. Transcriptome, surface proteome, and immunoreceptor sequencing data were generated on 580,040 high-quality cells, derived from 187 samples from 61 patients. For a subset of individuals, we obtained samples before and during infection, allowing intra-individual comparison. Longitudinal profiling demonstrated distinct temporal gene expression trajectories in severe/critical versus mild/moderate COVID-19. We identified a population of transcriptionally distinct monocytes that emerged in peripheral blood following glucocorticoid treatment. Evaluation of clonal T cell dynamics showed that the fastest expanding clones were enriched in known SARS-CoV-2-specific sequences and shared across multiple patients. Comparison with external datasets revealed upregulation of immune cell TGF-β pathway expression in ESKD, irrespective of COVID-19 status. Our data delineate the temporal dynamics of the immune response in COVID-19 in a high-risk population.

## Highlights

• Longitudinal immune gene expression profiles vary in mild versus severe COVID-19
• Interferon response gene upregulation occurs early and is greater in severe COVID-19
• Expanded clones carry SARS-CoV-2-specific TCRs and form MHC-restricted motifs
• A distinct population of monocytes emerges following glucocorticoid treatment

## Graphical abstract

**Figure:**
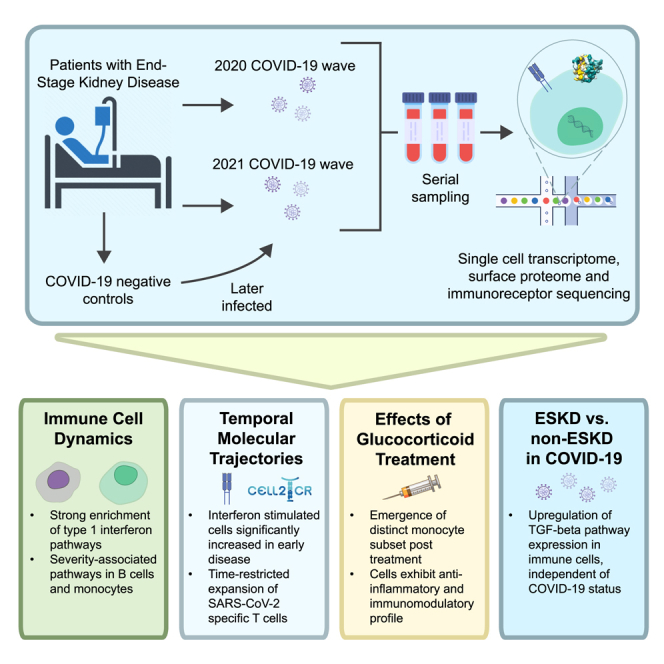

## Teaser

Stephenson et al. combine Cellular Indexing of Transcriptomes and Epitopes by Sequencing (CITE-seq) and VDJ sequencing to longitudinally profile the immune response to SARS-CoV-2 in patients with end-stage kidney disease. They demonstrate divergent immunological profiles in severe versus mild COVID-19, the emergence of transcriptionally distinct monocytes following glucocorticoid treatment, and SARS-CoV-2-specific T cell clones shared between multiple patients.

## Introduction

COVID-19, caused by the SARS-CoV-2 virus, displays marked clinical heterogeneity, varying from minimal symptoms to fatal disease. This variation in outcome is not random; severe or fatal COVID-19 disproportionately affects certain strata of the population. Demographic risk factors for severe COVID-19 include older age, male sex, and non-White ethnicity. Underlying medical conditions also impact the risk of severe COVID-19. End-stage kidney disease (ESKD) is one of the strongest risk factors for severe COVID-19, with a UK population-scale study estimating a hazard ratio for death of 3.7.^1^ There is, therefore, a need for research focusing on patients with ESKD and other high-risk groups.

A central feature of the pathophysiology of severe COVID-19 is an excessive host inflammatory response leading to tissue injury. Autopsies revealed an accumulation of activated immune cells but little or no active virus.^2^ Severe disease is characterized by excess circulating monocytes, neutrophils, and myeloid progenitors and elevated pro-inflammatory cytokines and chemokines, which contribute to endothelial damage and the formation of microthrombi. The importance of the host immune response is underscored by the efficacy of therapies targeting inflammation. Glucocorticoids, which have pleiotropic effects on inflammatory pathways, and targeted inhibition of the interleukin-6 (IL-6) signaling pathway both reduce mortality in COVID-19.^3^^,^^4^^,^^5^

ESKD is defined as irreversible loss of renal function, with a glomerular filtration rate of <15 mL/min/1.73 m^2^, that is fatal without dialysis or transplantation. In addition to loss of glomerular filtration, ESKD is a systemic disease associated with profound changes in hormonal, cardiovascular, and hematopoietic function.^6^ Such disturbances of normal physiology are also associated with immune dysfunction, and patients with ESKD have both increased susceptibility to infection and impaired vaccination responses.^7^^,^^8^^,^^9^^,^^10^ Despite impaired adaptive immune responses, ESKD is also characterized by a chronic pro-inflammatory state.^6^ Thus, patients with ESKD may be at high risk of complications of SARS-CoV-2 due to their preponderance of cardiometabolic risk factors, as well as both impaired immunity and a pro-inflammatory state. An outstanding question is whether patients with ESKD mount a distinct immunological response to SARS-CoV-2 that drives their susceptibility to severe COVID-19. Furthermore, the need to attend medical facilities for regular hemodialysis, regardless of infection with SARS-CoV-2, provides an opportunity to evaluate the temporal dynamics of the host immune response through serial sample collection in both inpatient and outpatient settings.

Here, we longitudinally profile the immune cellular landscape at single-cell resolution using multi-omics technologies during COVID-19 in the context of ESKD. Uniquely, we collected samples from the same set of individuals before and during SARS-CoV-2 infection. Our data elucidate the temporal dynamics of COVID-19 infection in a clinically vulnerable group.

## Results

### Longitudinal immune cell profiling in ESKD patients with COVID-19

**Figure 1:**
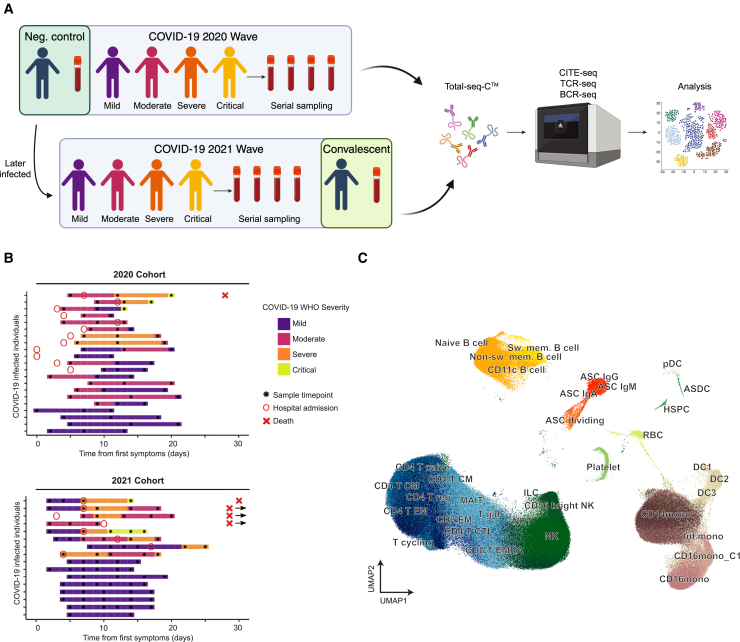
Study overview (A) Schematic of the study design. Neg. control = negative control (i.e., COVID-19^−^ patient with ESKD). Created using Biorender.com. (B) Timing of blood sampling in relation to COVID-19 onset. Colors indicate COVID-19 severity in a given individual over time. “X” with an adjacent arrow indicates death during the hospital admission occurring at >30 days (C) Uniform manifold approximation and projection (UMAP) showing the major cell-type annotations of B, myeloid and progenitor, and T cells, respectively. ASC, antibody-secreting cell; ASDC, Axl Siglec dendritic cell; CM, central memory; CTL, cytotoxic T lymphocyte; EM, effector memory; EMRA, terminally differentiated effector memory T cell; ILC, innate lymphoid cell; int, intermediate; MAIT, mucosal-associated invariant T cell; mono, monocyte; sw mem, switched memory; T g/d, gamma delta T cell.

We performed longitudinal blood sampling of peripheral blood mononuclear cells (PBMCs) from ESKD patients with COVID-19. Patients were recruited from a single center in London, UK, during two distinct waves of COVID-19. The first cohort (2020 Cohort/Wave 1) (*n* = 21) were recruited in April–May 2020, during the initial phase of the pandemic and before the advent of vaccination. This cohort consisted of ESKD patients with COVID-19, including both inpatients and outpatients, with a spectrum of COVID-19 severity from mild to critical (Figure 1A; Table S1). Following COVID-19 diagnosis, serial blood sampling was performed over the course of the illness (Figure 1B). In addition, we contemporaneously recruited ESKD patients who did not have COVID-19 to provide an appropriate control group (COVID-19 negative; hereafter, COVID-19^−^). This group was well matched in terms of age, sex, and ethnicity (Table S1).

The second cohort (2021 Cohort/Wave 2) (*n* = 16) consisted of ESKD patients with COVID-19, sampled between January and March 2021 (when the Alpha variant was the predominant SARS-CoV-2 variant in the United Kingdom). Again, serial blood sampling was performed during the acute illness. These patients were specifically re-recruited as they had been recruited the previous year as part of the COVID-19^−^ control group for the 2020 Cohort. Thus, for the 2021 Cohort we had matched samples from pre-infection (in 2020; pre-infection data available for 13 of 16 individuals) and during acute COVID-19 (in 2021), enabling intra-individual analysis. In addition, for a subset of the 2021 Cohort we collected a convalescent sample approximately 2 months after infection (*n* = 10) (Figures 1A and 1B). For the purposes of analysis, COVID-19^−^ samples from 2020, which had paired COVID-19 positive (hereafter, COVID-19^+^) samples from 2021 (from *n* = 13 patients), were analyzed together in the Wave 2 analysis and were excluded from the control group for Wave 1 to avoid sharing control samples across the two analyses, leaving 24 COVID-19^−^ samples in the Wave 1 analysis.

To assess cellular and molecular changes at single-cell resolution, we performed Cellular Indexing of Transcriptomes and Epitopes by Sequencing (CITE-Seq) of PBMCs with matched T cell receptor sequencing (TCR-seq) and B cell receptor sequencing (BCR-seq) (Figure 1A; Table S2). Following quality control steps, the dataset consisted of 580,040 cells, representing 187 samples from 61 patients. For initial cell-type annotation, we separated the data into three broad cell-type categories: (1) B cells, (2) T cells and innate lymphocytes, and (3) myeloid and non-immune hematopoietic cells (Figure 1C). Using semi-automatic cell-type annotations with CellTypist,^11^ COVID-19 reference atlases,^12^^,^^13^ and canonical marker genes, we identified 46 cell types (Figures 1C and S1A–S1D). These comprised known subtypes of monocytes (classical CD14^+^ monocytes, non-classical CD16^+^ monocytes, and intermediate CD14^+^CD16^+^ monocytes) and dendritic cells (DCs), plus sub-populations displaying an interferon (IFN)-stimulated signature^13^ and complement-expressing CD16^+^ monocytes that we have described previously (Figures 1C and S1B).^12^ Within the B cell compartment, we leveraged the availability of paired single-cell BCR-seq and CITE-seq data to detect eight sub-populations (Figures 1C and S1C). Similarly, using TCR-seq and CITE-seq data, we detected 22 clusters encompassing T cells, natural killer (NK) cells, and innate-like lymphocytes (Figures 1C and S1D).

### Altered cellular and transcriptomic profiles in ESKD patients with COVID-19

**Figure 2:**
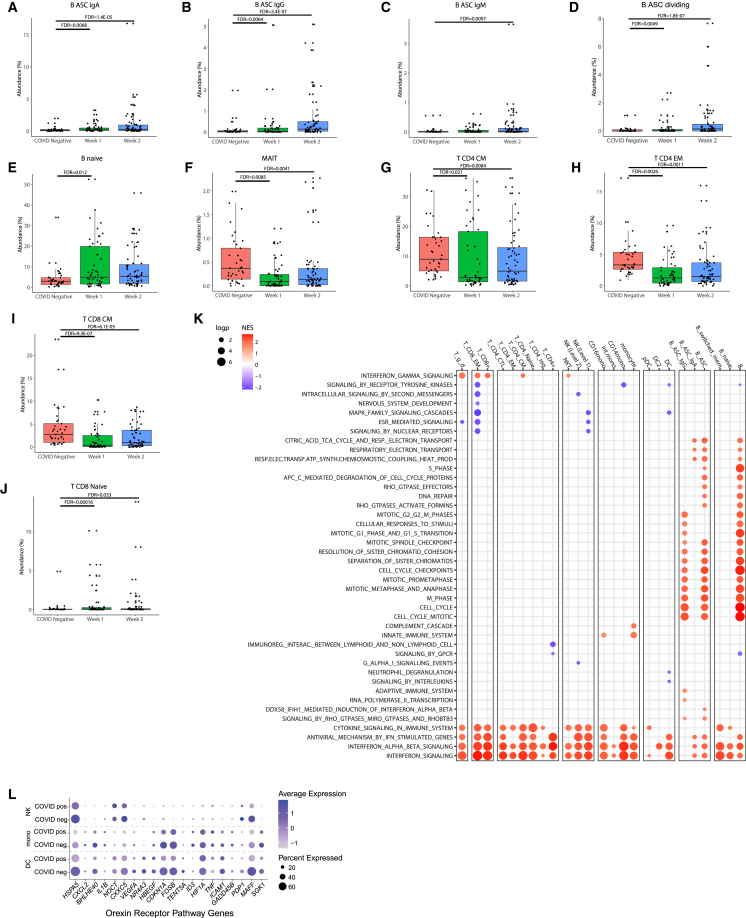
Cell-type abundance and differential gene expression and pathway analysis comparing COVID-19 positive versus COVID-19 negative ESKD patients (A–J) Bar charts displaying cell proportions that significantly changed in week 1 or 2 of COVID-19 infection compared to a control group of COVID-19^−^ ESKD patients. FDR-adjusted *p* values, with two significant digits shown. (K) Heatmap of gene expression pathways significantly (FDR <0.05) associated with COVID-19 positivity. Pathways were defined using the Reactome database. ELEC., electron; NES, normalized enrichment score; PROD., production; RESP., respiratory; TRANSP., transport. Log-p = −log10 BH-adjusted *p* value. (L) Dot plot displaying the expression of the leading-edge subset of genes that contributed to the term “orexin receptor pathway” for COVID-19^+^ and COVID-19^−^ ESKD patients. Mono = monocytes.

To evaluate changes in peripheral immune cellular proportions in COVID-19, we compared samples from COVID-19^+^ ESKD patients to those from COVID-19^−^ ESKD patients, stratifying the analysis according to whether samples were taken during the first or second weeks of COVID-19 (“week 1” and “week 2”). In week 1, there were increases in the proportion of naive CD8^+^ T cells and naive B cells compared to COVID-19^−^ samples. The proportions of antibody-secreting B cells (B-ASC) in PBMCs were increased in COVID-19^+^ samples in both weeks 1 and 2 (Figures 2A–2J and S2; Table S3), consistent with the development of an adaptive immune response. This effect was seen across multiple subclasses of ASC (immunoglobulin G [IgG], IgA, and IgM) as well as ASC with the transcriptional signature of dividing cells (B-ASC dividing). Similar findings have been observed in other studies of COVID-19 in non-ESKD populations.^12^^,^^13^ By contrast, there were decreases in the proportions of mucosal-associated invariant T cell (MAIT) cells, central memory CD4^+^ T and CD8^+^ T cells (T CD4 CM and T CD8 CM), and CD4^+^ effector memory cells (T CD4 EM). Decreases in certain T cell subsets in peripheral blood as well as generalized lymphopenia has been previously described,^12^^,^^13^^,^^14^ perhaps reflecting the migration of cells from blood to tissues.

To assess COVID-19-associated immune cell transcriptomic changes in ESKD, we performed differential gene expression analysis comparing COVID-19^+^ and COVID-19^−^ samples within each cell type (Table S4), using linear mixed models (LMMs) to account for non-independence of serial samples from the same individual. Gene set enrichment analysis was performed to identify the biological pathways implicated by the differentially expressed genes (DEGs; Table S5). In the 2020 Cohort, the most prominent finding was an enrichment of IFN-α and -β response pathways across a broad range of innate and adaptive immune cells (Figure 2K; Table S5). B cells exhibited the greatest number of significantly enriched pathway terms, totaling 240 pathways (Table S5). Many of these contained genes related to the cell cycle and DNA repair, likely reflecting the strong B cell proliferative response involved in initiating adaptive immunity to SARS-CoV-2. Similarly, there was an upregulation of genes relating to protein translation and post-translational modification, likely reflecting the generation of an antibody response. Many of the B cell-associated pathways were also enriched in B-ASC. Examining other cell types, we identified 19 enriched pathways in monocytes, 17 in NK cells, 15 in DCs, 8 in CD4^+^ T cells, and 7 in CD8^+^ T cells (Figure 2J; Table S5). Some pathways were significantly associated with COVID-19 across multiple cell types. For example, we observed a significant negative enrichment of the orexin receptor pathway across multiple innate immune cell types, including monocytes (Benjamini-Hochberg-adjusted *p* value [P_BH_] 2.36 × 10^−5^), DCs (P_BH_ 2.01 × 10^−8^), and NK cells (P_BH_ 0.014) (Table S5). The leading-edge genes that contributed to this term included many genes involved in the cellular stress response (Figure 2L). Analysis of monocyte subsets using higher-resolution annotation revealed enrichment of the pathway specifically in classical CD14 monocytes but not in intermediate and non-classical CD16 monocytes, suggesting the former was driving the signal. In the smaller 2021 Cohort, where we had paired pre-infection and infection samples from the same individuals, we replicated the findings of significant enrichment of the orexin receptor pathway' in CD14^+^CD16^−^ monocytes and NK cells but not in DCs (Table S5).

### Immune cell transcriptomic correlates of COVID-19 severity in ESKD

**Figure 3:**
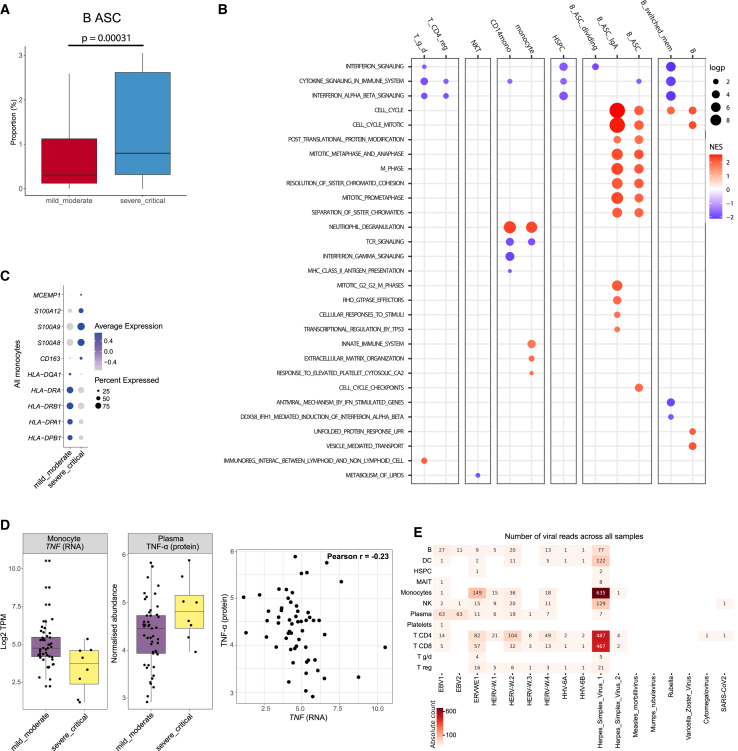
Cellular and transcriptomic changes associated with COVID-19 severity in ESKD (A) Proportion of antibody-secreting B cells in mild/moderate versus severe/critical COVID-19. BH-adjusted *p*-value 0.00031 (linear mixed model). (B) Heatmap of pathways significantly (FDR <0.05) associated with COVID-19 severity (Reactome database). (C) Expression of selected genes associated with COVID-19 severity in total monocytes. (D) Left: monocyte *TNF* mRNA expression (single-cell RNA-seq dataset). TPM, transcripts per million. Center: plasma TNF-α protein abundance (Olink immunoassays). *n* = 57 samples from 21 individuals with both RNA and plasma protein levels measured. Right: correlation between monocyte *TNF* gene expression and TNF-α plasma protein levels. (E) Heatmap displaying the number of viral reads from different viral genomes, including SARS-CoV-2 (NC_045512.2), detected across cell types.

We next assessed molecular and cellular changes associated with COVID-19 severity at the time of blood sampling. Comparison of samples taken from patients at the time of severe or critical COVID-19 (hereafter, severe/critical, *n* = 56) to those taken at the time of mild or moderate disease (hereafter, mild/moderate, *n* = 84) revealed that the proportion of B-ASC cells was increased in the severe/critical group (Figure 3A; Table S6). We then performed differential gene expression within each cell type, again comparing samples taken at the time of severe/critical COVID-19 to mild/moderate COVID-19 (Table S7). Pathway enrichment analysis was performed on genes associated with COVID-19 severity (Figure 3B; Table S8), revealing 86 pathways associated with COVID-19 severity. The majority of these were in the B cell (35) or monocyte (29) compartment. 11 pathways were associated with severity in NK cells, 5 in gamma delta cells, 2 in CD4^+^ T cells, and 0 in CD8^+^ T cells. Similarly, at the gene level, 205 genes were associated with COVID-19 severity; 125 of these were in all monocytes or CD14^+^CD16^−^ classical monocytes and 21 were in B cell subsets (Table S8).

In B cells, ASCs, and ASCs that produce IgA, pathways relating to cell division were enriched in severe/critical COVID-19, likely representing a more marked adaptive immune response. In dividing antibody-secreting cells (B_ASC_dividing) and switched memory B cells, IFN signaling pathways were increased in severe/critical COVID-19 (Table S8). In monocytes, Kyoto Encyclopedia of Genes and Genomes (KEGG) pathway terms associated with COVID-19 severity included “asthma,” “graft-versus-host disease,” “leishmania infection,” and “allograft rejection” (Table S8). Many genes in these pathways were downregulated in severe/critical relative to mild/moderate COVID-19, and the enrichment of these pathways is driven, in part, by the high representation of *HLA* genes. This is consistent with the downregulation of major histocompatibility complex (MHC) molecules on antigen-presenting cells in severe COVID-19 that has been reported previously.^15^ We observed downregulation of *HLA-DPB1*, *HLA-DPA1*, *HLA-DRB1*, *HLA-DRA*, and *HLA-DQA1* in total monocytes (Figure 3C). This was accompanied by the upregulation of *CD163* as reported in other studies.^15^^,^^16^^,^^17^ We also observed the upregulation of genes previously associated with severity such as *S100A8*, *S100A9*, *S100A12*, and *MCEMP1* (Figure 3C).^18^ In all monocyte subsets, the most strongly differentially expressed gene between mild/moderate and severe/critical samples was tumor necrosis factor (*TNF*), encoding TNF-α (*p* = 6.4 × 10^−116^) (Table S7). Unexpectedly, given its pro-inflammatory effects, *TNF* gene expression was lower in severe/critical COVID-19. We hypothesized that this might be as a result of negative feedback from elevated TNF-α at the protein level. We therefore analyzed TNF-α protein levels using Olink immunoassays in plasma from the same set of blood samples. This revealed higher plasma TNF-α protein in samples taken at the time of severe/critical COVID-19. There was a weak negative correlation (Pearson *r* −0.23) between plasma TNF-α protein and monocyte *TNF* gene expression, demonstrating an uncoupling of plasma protein and gene expression levels (Figure 3D).

Another pathway enriched in severe/critical COVID-19 in CD14^+^ monocytes was “neutrophil degranulation” (Table S8). Further examination of this pathway association revealed *PLAC8* as a leading-edge gene and that *PLAC8* was significantly upregulated in severe disease (Figure S3E; Table S7). *PLAC8* overexpression *in vitro* has been previously shown to make lung cells more permissive for SARS-CoV-2 infection.^19^ Furthermore, a genome-wide CRISPR knockout screen identified PLAC8 as an essential factor for infection with a different coronavirus, swine acute diarrhea syndrome coronavirus.^20^ While SARS-CoV-2 predominantly infects epithelial cells, detection has been reported in macrophages and T cells.^21^ We therefore considered whether the association between increased expression and severe COVID-19 reflected a causal role for PLAC8 in the pathogenesis of COVID-19 severity mediated through increased viral entry. To evaluate this, first, we leveraged our pre-infection samples and tested whether *PLAC8* expression in CD14 monocytes prior to infection was associated with subsequent peak illness severity during COVID-19. We found no association between pre-infection *PLAC8* gene expression and subsequent COVID-19 severity (Figure S3F). Second, we examined SARS-CoV-2 viral load in immune cells, including monocytes. This revealed no significant viral load in immune cells (Figure 3E), providing no evidence to support a role for PLAC8 in mediating viral entry in monocytes or other peripheral blood immune cells *in vivo*. Third, we performed Mendelian randomization (MR), an analytical technique that leverages naturally occurring genetic variation to evaluate whether *PLAC8* gene expression plays a causal role in COVID-19 severity. Using two-sample MR, we found no significant association between genetic variants that influence *PLAC8* gene expression and COVID-19 severity (Figure S3G). These lines of data do not support a role for *PLAC8* gene expression as a causal factor in COVID-19 severity.

In NK cells, we identified COVID-19 severity-associated genes relating to the TLR4 and TLR9 signaling pathways and to the oncostatin M pathway (Tables S7 and S8). We previously reported the upregulation of plasma protein levels of oncostatin M in severe COVID-19.^22^ This cytokine is known to regulate IL-6 and GM-CSF production, which have been previously implicated as drivers of severe COVID-19.^23^

### Temporal gene expression trajectories vary according to disease severity

**Figure 4:**
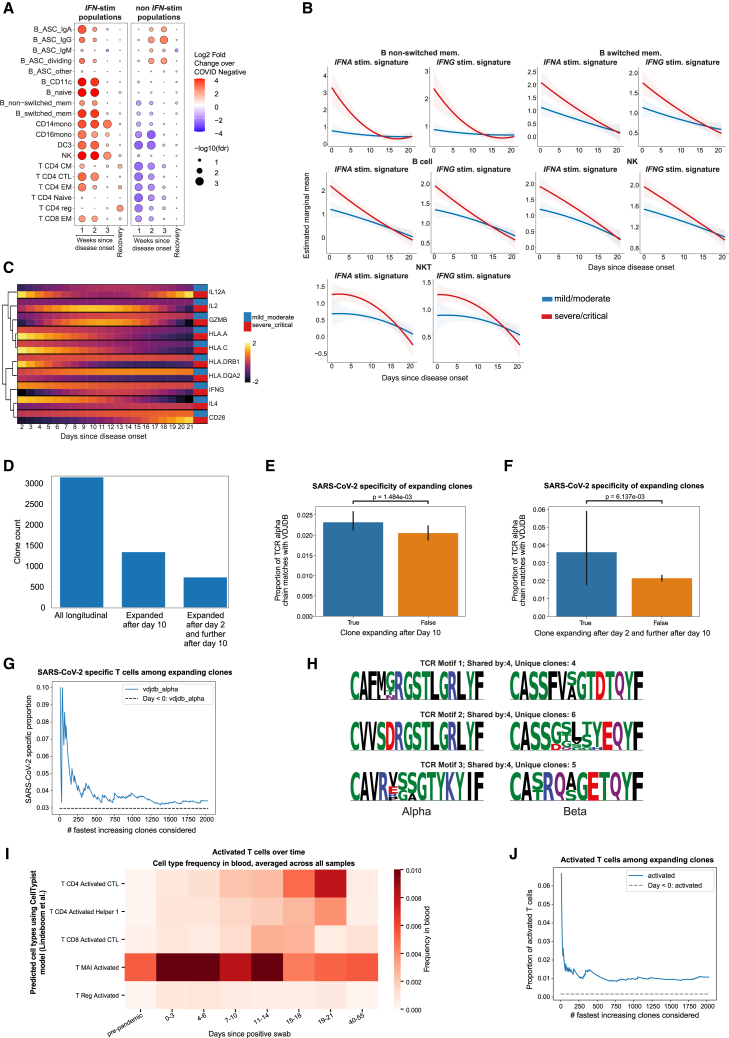
Longitudinal gene expression and TCR trajectories (A) Temporal cell-type abundance changes in COVID-19 over time, stratified by whether the cells exhibit an interferon (IFN)-stimulated state. Log_2_ fold change relative to COVID-19^−^ samples. Significance measured by the local true sign rate with FDR control. Only cell types that have an IFN- and non-IFN-stimulated counterpart are shown. Sample *n*: 37 COVID-19^−^, 138 COVID-19^+^, 10 recovery. (B) Temporal changes in IFN pathway gene expression, stratified by peak illness severity. Estimated marginal mean (line) and 95% confidence intervals (shade). Cell types with significant time × severity interaction from LMM shown. *n* = 139 COVID-19^+^ samples. (C) Heatmap displaying 10 genes from multiple KEGG immunological disease-associated pathways that had a significantly different temporal profile in mild versus severe COVID-19 (LMM, FDR < 0.05) in CD14 monocytes. Color indicates LMM estimated marginal means over time, stratified by patient group (*n* = 130 samples from 37 individuals). Genes selected to represent different temporal dynamics and clustered based on the temporal profile of the discordance between mild/moderate and severe/critical disease. For (A)–(C), time since onset of disease represents either time since display of first symptom or positive test (whichever is earliest). (D) Absolute numbers of clones considered for longitudinal analysis and expanded clone counts (from *n* = 139 COVID-19^+^ samples, and the same samples analyzed for (E)–(J). (E) Proportion of SARS-CoV-2-specific clones among all clones, stratified by whether the clone expanded after day 10 following positive PCR test. Specificity defined as a perfect match with a TCR alpha chain from the SARS-CoV-2 database VDJDB. Two-sided Mann-Whitney test. (F) As for (E) but stratifying by whether a clone was expanded after day 2 and further after day 10. (G) SARS-CoV-2-specific clone proportion among fastest-increasing clones. Clones sorted by decreasing expansion magnitude after day 10 following positive PCR test. Dashed line: baseline of matches with database from pre-pandemic samples. (H) Sequence logos of three most shared paired-chain TCR motifs, showing the number of individuals and number of unique clones sharing the motif. Letter height indicates frequency of amino acid (aa) at that position across T cells pertaining to the motif. Each aa is colored by side chain chemistry: acidic (red), basic (blue), hydrophobic (black), neutral (purple), and polar (green). (I) Distribution of predicted activated T cells across days since positive swab result. T cell-type frequency averaged per sample and aggregated across time points. Cell states predicted using Celltypist. (J) Activated T cell state proportion among fastest-increasing clones. Clones sorted by decreasing expansion magnitude pre-/post-day 10 following positive PCR test. Dashed line: baseline proportion of activated T cells from pre-pandemic samples.

The host response to infection is a dynamic process involving both the innate and adaptive immune systems. To understand these temporal dynamics in COVID-19, we performed longitudinal analysis of our multi-omics data. Cell-type composition analysis revealed that most cell subtypes displaying an IFN-stimulated gene expression signature were significantly increased within the first week following symptom onset and then gradually reduced over time (Figure 4A). We observed significant increases in the relative abundance of IFN-stimulated cell types persisting into weeks 2 and 3 following symptom onset (switched memory B cells, CD14 and CD16 monocytes, DC3, NK, T CD8^+^ effector memory [EM], T CD4^+^ cytotoxic T lymphocyte, and EM cells). As expected from a viral airway infection, compositions of antibody-secreting B cells, predominantly of class-switched (IgG and IgA) antibody isotypes, were increased already in the first week after onset of disease, persisting for up to 3 weeks in both IFN-stimulated and non-stimulated states. In convalescent samples taken approximately 2 months after the acute infection, we observed an enrichment of some IFN-stimulated cell types, including CD4^+^ regulatory T cells, T CD4^+^ EM and T CD4^+^ CM. Almost all non-IFN-stimulated states returned to pre-infection levels in convalescent samples (Figure 4A).

We next assessed the temporal patterns of gene expression changes during COVID-19 in patients with ESKD and how these vary according to overall clinical course (defined by peak illness severity, binarized as mild/moderate or severe/critical). To achieve this, we performed longitudinal modeling using an LMM with a time × peak severity interaction term. To reduce dimensionality, we analyzed genes grouped together as modules according to pathway terms, using the Hallmark, Reactome and KEGG databases. A pathway with a significant time × severity interaction indicates that the pathway has a different temporal profile in mild/moderate versus severe/critical COVID-19. Our analysis revealed 177 pathways with significant (false discovery rate [FDR] <0.05) time × severity interactions (Table S9). Notably, the majority of the significant time × severity interactions were in B cells, accounting for 143 of the 177 significant pathways. The 20 pathways with the most significant time × severity interactions were predominantly in B cells and monocytes. The two pathways showing the most significant time × severity interaction were the IFN-α and IFN-γ response in non-class-switched memory B cells. Significant time × severity interactions for these pathways are also observed in B cells, switched and non-switched B memory cells, NK cells, and NKT cells (Table S9). These results reflected quantitative differences in the temporal gradient of the IFN pathway response, with more severe COVID-19 disease showing higher IFN pathway response early in disease and a steeper decline over time (Figure 4B).

In both CD14^+^ monocytes and B cells we found significant time × severity interactions for “allograft rejection” pathways and in CD14 monocytes for “graft-versus-host disease,” “asthma,” “type 1 diabetes,” and “systemic lupus erythematosus.” Examination of the genes that make up these pathways revealed that these signals were largely driven by distinct temporal patterns of *HLA* expression. In individuals with a severe/critical clinical course, we observed steep downregulation of *HLA* class II gene expression over time, compared to either a relatively flat or mild upregulation in individuals with a more benign course. *HLA* class I gene expression was higher in early disease in individuals with severe/critical disease than in mild disease but fell further in late disease (Figures 4C and S4A). Other pathways with significant time × severity interactions included “KRAS signaling” and “MYC targets” in B cells, likely reflecting time-dependent changes in their proliferation during infection that vary according to severity (Table S9).

These results illuminate how modeling the temporal component provides additional insights by identifying time-dependent severity associations with gene expression that are not apparent in single time point cross-sectional analyses. Transcriptomic changes are dependent both on time and severity and the interplay of two, underscoring the importance of serial sampling in gaining a complete picture of the host immune response in COVID-19.

### Longitudinal TCR dynamics

Given the importance of T cells in antiviral adaptive immunity, we evaluated clonal T cell dynamics during SARS-CoV-2 infection. The longitudinal study design and single-cell resolution enabled us to determine paired-chain clones that expanded over the course of COVID-19. A total of 3,137 unique TCR clones that appeared in two or more serial samples from the same patient were used to quantify clonal expansion. To increase the probability of identifying TCR clones specific to SARS-CoV-2, we focused on clones that were not present in pre-infection samples, thereby limiting the presence of cross-reactive or bystander T cells. We found that 42% of clones sampled longitudinally had increased clonal frequency following day 10 after a positive SARS-CoV-2 nasal swab; 23% showed a marked expansion with increase after day 2 of the positive swab and increased further after day 10 (Figures 4D, S4B, and S4C). To investigate whether these clonal expansions were directed against SARS-CoV-2, we cross-referenced SARS-CoV-2-specific TCR sequences from the VDJDB database^24^ and measured the overlap with clones identified in more than one serial sample within an individual. Clones expanding after day 10 were significantly enriched in SARS-CoV-2-specific TCR alpha chains (*p* = 0.0014, two-sided Mann-Whitney test; Figure 4E) compared to their non-expanding counterparts, while those fulfilling the stricter dual criteria above had an almost 2-fold increase in antigen-specific TCR alpha chains (*p* = 0.0061; Figure 4F). Examination of the relationship between magnitude of expansion of the longitudinally identified clones and SARS-CoV-2 specificity showed that the fastest-expanding clones had the highest proportion of SARS-CoV-2-specific TCR alpha chains (Figure 4G). This SARS-CoV-2 specificity estimate is likely a lower bound to the true number, as experimental data from the database are based on assays with many fewer SARS-CoV-2 peptides than the number of naturally occurring viral antigens. Thus, of the expanding sequences that we recovered that do not match the database, more are likely to be virus specific. A caveat to this analysis is that we did not observe this effect for SARS-CoV-2-specific TCR beta chains (*p* = 0.85; Figures S4D and S4E). This may be caused by their larger sequence diversity, making it harder to find matching sequences in sparse single-cell data compared to the more public TCR alpha chains, but we cannot exclude the possibility of non-specificity of the expanding clones, for example, due to bystander activation.

We searched for patterns in the TCRs of expanding clones that might be shared across individuals. We inferred TCR motifs across the expanded clones (47,443 unique T cell clones, excluding MAIT cells) and found 99 public TCR motifs, defined as a group of clonotypes with sufficient sequence similarity to likely recognize the same epitope and that was found in two or more patients. Moreover, six TCR motifs were shared between three patients and three TCR motifs between four patients (Figure 4H). This scenario is highly unlikely for randomly sampled TCR clones and provides evidence of strong selective pressure on the adaptive immune response to a common pathogen, illustrated by the fact that TCR motif inference on the entire naive T cell compartment of all patients (37,955 unique clones), to provide an unbiased repertoire control, yielded only a total of 5 public TCR motifs. To investigate whether shared TCR motifs reflected MHC restriction, we inferred the *HLA* genotypes of study participants from the raw single-cell sequencing reads (Table S10), and we examined whether patients with a given TCR motif also shared *HLA* alleles. For the three TCR motifs common to four patients (Figure 4H), we identified *HLA-DPA:01:03* as shared among all patients with a given motif for the α subunit of the MHC molecule. Concerning the β subunit, TCR motif 1 (Figure 4H) is likely either *DPB1∗02:01* or *DPB1∗04:01* restricted, since three out of four patients shared these. TCR motifs 2 and 3 are both predicted to be *DPB1∗04:01* restricted, with all or three out of four patients matching this *HLA* type, respectively. Thus, all of the TCR motifs are likely to have MHC class II interaction partners that are shared across patients. The three TCR motifs shown in Figure 4H were derived from T CM, EM, and cytotoxic CD4^+^ T cells, and MHC class II restriction is consistent with the mode of antigen presentation to CD4^+^ T cells. No MHC class I restriction was observed for these TCR motifs. Notably, T cells from TCR motifs that were shared across three or more patients were enriched for IFN-stimulated states compared to those that were not shared (*p* = 0.0057, chi-squared test). In addition, naive T cells were virtually absent from TCR motifs that were shared across 2 or more patients (2 of 1,087 T cells).

Since we had recruited patients during two distinct phases of the pandemic, we hypothesized that certain TCR motifs might be specific to a particular viral strain and exhibit sharing only across patients from the same cohort (i.e., sampled in 2020 or 2021). Of the 99 public TCR motifs, 64% were specific to patients from one cohort, including TCR motifs 1 and 3 (Figure 4H) that were each shared across 4 patients. Furthermore, 18% of public motifs contained at least one SARS-CoV-2-specific TCR sequence, underscoring the utility of this approach to analyze the antigen-specific response. Our findings are in line with evidence from a recent SARS-CoV-2 human challenge study (SHCS),^25^ which entailed deliberate infection of healthy individuals with SARS-CoV-2 and showed that the antigen-specific response included convergent paired-chain immune receptor motifs. We thus replicate the SHCS results in the context of natural infection and in a larger cohort comprising a clinically vulnerable group consisting of older individuals with underlying comorbidities, which included cases of severe/critical COVID-19.

We next investigated for the presence of time-restricted, activated T cell types described in the SHCS, where activated T cell states were found to be indicative of *de novo* T cell activation and harboring SARS-CoV-2-specific TCR sequences. Automated cell state annotation revealed 1,927 activated T cells in our dataset, spanning the CD4^+^, CD8^+^, regulatory, and MAIT cell compartments and found among 58 ESKD patients (Figure S4F). When computing the cell-type frequency per sample and aggregating across time points, we observed a lack of predicted activated T cells in pre-pandemic as well as convalescent COVID-19 samples (Figure 4I). While MAIT cells and regulatory T cells showed relative enrichment during the first week after positive PCR test, most activated CD4^+^ and CD8^+^ T cells appeared only after 10 days. All predicted activated T cell types remained detectable 3 weeks after positive PCR test but had mostly disappeared again by the time convalescent samples were taken, highlighting the transient nature of these cell states. Activated T cells were further overrepresented among the most expanded clones (Figure 4J). This is in line with results from the SHCS, where activated MAIT cells could be detected as early as 3 days after exposure to the virus, and circulating activated T cell abundance peaked 10–14 days after exposure to the virus, with return to baseline after 28 days.^25^

### Glucocorticoids induce dexamethasone-related monocytes in COVID-19

By the time of recruitment of the 2021 Cohort, glucocorticoid administration with dexamethasone had become standard practice in the UK for severe COVID-19 following randomized clinical trials demonstrating that it reduced mortality in patients with COVID-19 requiring supplemental oxygen.^3^ Glucocorticoids have broad immunosuppressive effects through several different mechanisms, including inhibiting the release of proinflammatory cytokines.^26^ *In vitro* experiments have suggested that monocytes and macrophages treated with glucocorticoids can exhibit both anti-inflammatory and inflammation-resolving properties.^27^ The effect of glucocorticoids on human immune responses at the single-cell level *in vivo* has not been studied. Of the 16 patients in the 2021 Cohort, 7 received steroid treatment (Table S1). Patients receiving glucocorticoids had a peak illness severity of severe or critical. This provided us with an opportunity to investigate the effects of steroids at the single-cell transcriptomic level over the course of their treatment.

**Figure 5:**
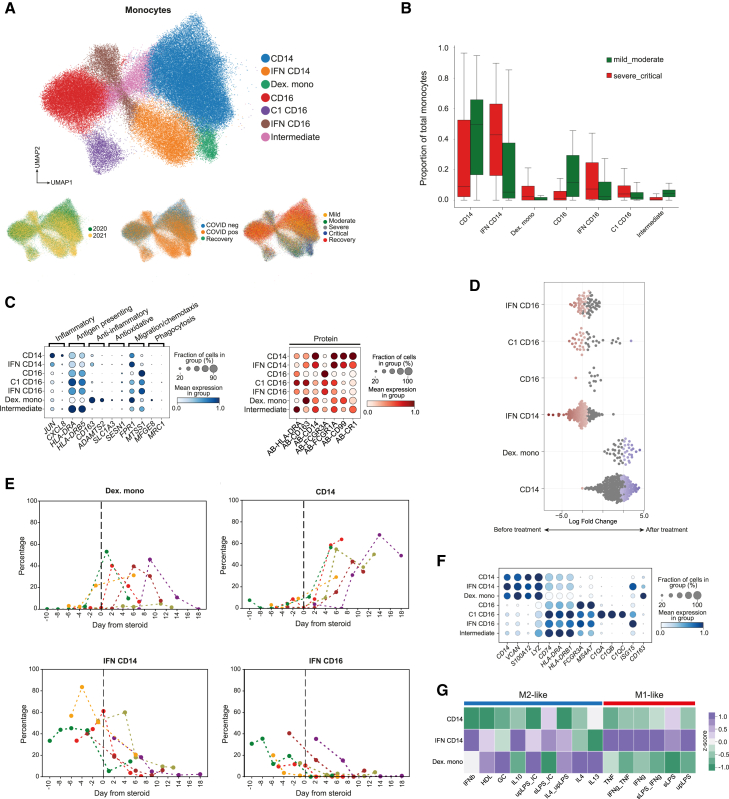
Dexamethasone treatment promotes a distinct monocyte subset (A) UMAPs displaying monocytes; colored by subset (top), patient cohort (bottom left), COVID-19 status (bottom center), and severity (bottom right). (B) Proportions of total monocytes stratified by COVID-19 severity. (C) Gene expression (left) and protein expression (right) across monocyte subsets. (D) Differential abundance of monocyte subsets for samples from patients given glucocorticoids pre- and post-treatment. (E) Intra-individual changes in monocyte subsets pre- and post-glucocorticoids. Line colors represent different patients. (F) Expression of monocyte marker genes across monocyte subsets. (G) Gene module scores for CD14 monocytes, IFN-stimulated CD14 monocytes and the dexamethasone-associated monocytes (dex. mono).

Evaluation and clustering of the cells in the monocyte compartment revealed the emergence of a distinct population of monocytes restricted to severe/critical COVID-19 and not present in samples from patients with mild/moderate COVID-19 or without COVID-19 (Figures 5A and 5B). Differential gene expression analysis between all subsets of monocytes showed that this population had transcriptional similarities with monocytes treated *ex vivo* with dexamethasone^28^ (Figure 5C). Compared to classical CD14 monocytes and IFN-stimulated CD14 monocytes, the dexamethasone-related monocytes (dex. monos) had lower expression of markers of inflammation such as *JUN* and *CXCL8*, as well as lower expression of antigen-presenting markers *HLA-DRA* and *HLA-DRB5*. Conversely, they showed higher expression of genes relating to anti-inflammatory actions (*CD163* and *ADAMTS2*), anti-oxidation (*SLC1A3* and *SESN1*), migration (*FPR1* and *MTSS1*), and phagocytosis (*MFGE8* and *MRC1*) (Figure 5C). Notably, these cells were present only in patients recruited in the 2021 Cohort, not the 2020 Cohort, suggesting they were a direct effect of glucocorticoid treatment and not a consequence of severe COVID-19 itself (Figure 5A).

We formally tested the effect of glucocorticoids on differential cell abundance across the monocyte clusters, accounting for time from infection.^29^ We noted that both CD14 monocytes and the dex. monos were significantly enriched after glucocorticoid treatment, and the IFN-stimulated CD16 monocytes, C1 CD16 monocytes, and IFN-stimulated CD14 monocytes were significantly enriched before treatment (Figure 5D). Using the longitudinal data from only the individuals who were given glucocorticoid treatment, we evaluated the percentage of different monocyte subsets prior to and in the days after treatment. We found that after glucocorticoid administration, there was a trend toward an increased abundance of the dex. monos and CD14 monocytes, while there was a decrease in both IFN-stimulated monocyte populations (Figure 5E). No trends were observed in other cell types (Figures S5 and S6).

The dex. monos displayed high RNA and protein expression of CD163 (Figures 5C and 5F), a scavenger receptor that is frequently used to mark “alternatively activated” or “M2”-like macrophages.^30^ These macrophages possess regulatory functions that can suppress immune responses and reduce inflammation.^31^ Macrophages treated with glucocorticoids have been shown to drive the polarization of macrophages toward an alternatively activated/M2-like phenotype.^32^ These findings prompted us to further assess transcriptional programs of the dex. monos. We performed pathway enrichment analysis on all monocytes based on 15 different macrophage stimulation signatures.^33^ The transcriptomic profiles of the dex. monos were most correlated with those of monocytes stimulated with IL-13, IL-4, ultra-pure lipopolysaccharide (LPS)+immune complex, and glucocorticoid stimulation, supporting the similarity of dex. monos to M2-like macrophages (Figure 5G). The temporal emergence of the dex. monos and their presence only in the 2021 Cohort imply that their emergence were driven by dexamethasone treatment rather than disease severity.

### Similar immune gene programs during COVID-19 in patients with or without ESKD

**Figure 6:**
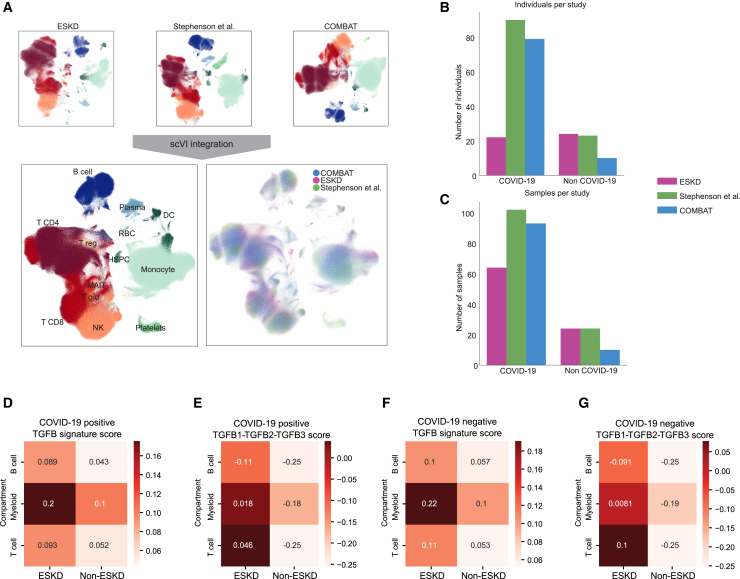
Comparison of COVID-19-associated transcriptomic changes in ESKD versus non-ESKD cohorts (A) UMAPs displaying major PBMC cell types for each of the datasets individually and after integration. (B) Number of individuals per study, separated by COVID-19 status. (C) As for (B) but showing the number of samples. (D) TGF-β signature score for COVID-19^+^ samples aggregated across cell compartments and stratified by ESKD status. (E) Combined score for *TGFB1*, *TGFB2*, and *TGFB3* genes, for COVID-19^+^ samples aggregated across cell compartments and stratified by ESKD status. (F) As for (D), but for COVID-19^−^ samples. (G) As for (E), but for COVID-19^−^ samples.

The increased prevalence of severe or fatal COVID-19 in patients with ESKD compared to the general population raises the question: is the host immune response to SARS-CoV-2 in ESKD distinct from that in non-ESKD? To assess this, we compared our COVID-19 ESKD single-cell multi-omics data to two previously published datasets generated from COVID-19 patients without ESKD^12^^,^^34^ (Figures 6A–6C). These two datasets included patients who were recruited at a time period similar to that of the ESKD 2020 Cohort. We integrated the three datasets using a variational autoencoder and inference approach and harmonized the cell-type annotations (Figure 6A) to enable cross-dataset comparison.

We analyzed the transcriptome of immune cell types, comparing samples from patients with COVID-19 and ESKD to those with COVID-19 without ESKD (Table S11). Pathway analyses showed that there was an increase in the gene expression program related to transforming growth factor β (TGF-β) signaling pathway across all cell lineages in ESKD patients, including the genes encoding TGF-β1, TGF-β2, and TGF-β3 (Figures 6D and 6E; Table S12). Comparison of COVID-19^−^ ESKD patients to COVID-19 healthy controls revealed the same pattern, indicating that this effect relates to ESKD independent of COVID-19 status (Figures 6F and 6G, shown in aggregate for each cell compartment, and STAR Methods). No other pathways were significantly enriched between ESKD versus non-ESKD in the context of COVID-19.

## Discussion

Here, we performed CITE-seq and immunoreceptor profiling to longitudinally profile the circulating immune cell changes associated with COVID-19 in the context of ESKD in two temporally distinct cohorts. A unique aspect of our study was the 2021 Cohort, where we obtained longitudinal PBMC samples from patients with COVID-19 who were originally sampled as COVID-19^−^ controls during 2020 but subsequently became infected during 2021. As a result, we were able to perform intra-individual analysis of the host immune cell PBMC transcriptome comparing pre-infection with acute infection, thus minimizing the impact of confounding factors. Another distinct feature of our study was the inclusion of patients of diverse ancestries.

We identified COVID-19-associated changes in the cellular composition of PBMCs in ESKD patients, including a decrease in the relative abundance of total monocytes and the subpopulations of CD14, CD16, and intermediate monocytes. This decrease in the relative numbers of circulating monocytes following infection was also observed in an experimental SHCS involving deliberate infection of healthy individuals with SARS-CoV-2.^25^ COVID-19 was associated with widespread transcriptomic changes in a wide variety of cell types. Many of these reflect the activation of inflammatory pathways, including the type I IFN pathway and cellular activation and proliferation. We integrated our data on COVID-19^+^ and COVID-19^−^ ESKD patients and performed comparisons with two other single-cell multi-omics studies that included hospitalized COVID-19^+^ non-ESKD patients and healthy controls. In ESKD, there was an increase in the gene expression program related to TGF-β signaling pathway but this effect was present irrespective of COVID-19 status. We did not observe any COVID-19-associated effects that were specific to ESKD.

Gene expression pathways associated with COVID-19 severity were particularly enriched in monocytes and B cells. In contrast, there was a paucity of significant pathway associations in T cells. In addition, severe COVID-19 was associated with a higher relative abundance of antibody-secreting B cells and with higher expression of genes involved in cell division. Multi-omics measurements allowed us to identify instances of negative correlation between immune cell gene expression and levels of the corresponding plasma protein. For example, in severe COVID-19, the most downregulated gene in monocytes was *TNF* (encoding TNF-α), yet conversely, TNF-α was significantly upregulated in the plasma from the same blood draw. Potential explanations for this uncoupling include negative feedback or that other cell types could be contributing to the circulating TNF-α pool (e.g., endothelial cells, tissue macrophages). This observation underlines the complementary value of combining multi-omics data, since plasma proteins reflect protein production by a wide variety of tissues other than blood cells.^35^ A caveat is that since our data are observational, we cannot determine whether elevated circulating TNF-α is a cause or a consequence of severe COVID-19.

Longitudinal analysis of changes in cell-type abundance showed a peak in cells showing an IFN-activated gene signature in the first week of illness followed by a waning, consistent with previous findings.^25^ Gene expression pathways that displayed distinct temporal profiles according to clinical severity were predominantly found in B cells and monocytes. Longitudinal analysis also revealed the time-restricted appearance and expansion of T cells with likely SARS-CoV-2 specificity. Leveraging the large number of longitudinal samples, we identified the emergence of public T cell clones with a restricted TCR repertoire that are shared across individuals. Cross-referencing the TCRs with SARS-CoV-2-specific databases, we found most matches among T cells strongly expand in the days following a positive PCR test and a significant enrichment of matches compared to pre-pandemic samples. Consistent with the role of both TCR chains together determining antigen specificity, which we were able to capture jointly using single-cell sequencing, expanding clones could further be grouped into shared TCR motifs with high sequence similarity in both chains. We inferred *HLA* genotypes of the entire cohort and used these to show MHC restriction of TCR motifs and predict MHC molecules presenting to T cells from shared TCR motifs. In addition, we found that a recently activated T cell phenotype is overrepresented in that same population. We expand on existing knowledge from the SHCS by analyzing a larger patient cohort, which included cases of severe disease, as well as replicating several key findings in the context of natural infection. In addition, we identified longitudinal expansion of some clones that are not recorded as SARS-Cov2 specific in the VDJDB database. These could be previously undescribed SARS-CoV2-specific T cells, given that we analyzed a large number of longitudinal samples that included patients with severe/critical disease and also diverse ancestry. An alternative explanation is that the expansion of some TCR clones was driven by bystander activation secondary to the inflammatory milieu.

We identified a distinct population of monocytes that emerged after glucocorticoid therapy. These were not observed in any patients in the 2020 Cohort, before the introduction of glucocorticoid therapy as standard of care, and their emergence in the 2021 Cohort occurred rapidly after glucocorticoid administration. These findings could have implications beyond COVID-19. Corticosteroids are used frequently to suppress inflammation, and they have pleiotropic effects on immunity that are not fully understood, despite their long-standing clinical use. Here, we demonstrated that glucocorticoids could promote the emergence of a transcriptionally distinct subpopulation of monocytes, although whether this relates to therapeutic benefit remains unclear. Our data support a recent report of a similar monocyte population with immunomodulatory functions in patients with COVID-19 (without ESKD) treated with dexamethasone.^36^ This study also described a glucocorticoid-specific reversal of a dysfunctional monocyte state in patients with severe COVID-19 but not in patients with fatal disease. An outstanding question remains the functional properties of the steroid-induced cells, such as their ability to traffic to tissues and modulate inflammatory responses.

### Limitations of the study

Our data are observational and thus cannot delineate whether changes in cell populations or gene expression are pathogenic drivers or downstream consequences of the systemic inflammatory response. Observational data are also vulnerable to the effects of confounding factors. Our use of paired pre-infection and infection samples in the analysis of the 2021 Cohort partially mitigates this, but unknowable confounders such as viral exposure at the time of infection may nevertheless impact the magnitude of the host immune response. In addition, we studied peripheral blood immune cells due to accessibility, but these may not always reflect those at the site of tissue inflammation. We did not have a comparator group of ESKD patients with another infection, so we cannot determine whether the changes we observed are specific to COVID-19. Finally, the primary data were from a single center study. While we used integration to allow cross-comparison with non-ESKD COVID-19 cohorts, there is a possibility of technical or clinical/biological differences between cohorts confounding the comparison of COVID-19 in ESKD to COVID-19 in non-ESKD. Of note, our cohorts contained both hospitalized patients and those managed in community, whereas the comparator studies exclusively involved hospitalized patients.

In summary, we characterized the longitudinal host immune response in COVID-19 in ESKD through multi-omics technologies. These data illuminate the temporal dynamics of the response to infection and how these diverge in mild versus severe disease.

## Resource availability

### Lead contact

Requests for further information and resources should be directed to the lead contact, James E. Peters (j.peters@imperial.ac.uk).

### Materials availability

This study did not generate new unique reagents.

### Data and code availability

De-identified patient single-cell count matrix and associated metadata are available at the COVID-19 Cell Atlas web portal as an h5ad file (on covid19cellatlas.org/index.patient.html, link for direct download at https://covid19.cog.sanger.ac.uk/eskd_covid19.h5ad). Original code has been deposited on Zenodo at https://doi.org/10.5281/zenodo.15358026. All are publicly available as of the date of publication. Any additional information required to reanalyze the data reported in this paper is available from the lead contact upon request.

## Acknowledgments

This work was funded by a UKRI-DHSC COVID-19 Rapid Response Rolling Call (grant no. MR/V027638/1) (to J.E.P.), funding from the 10.13039/501100023449UK Coronavirus Immunology Consortium, Wellcome Human Cell Atlas Strategic Science Support (grant no. WT211276/Z/18/Z), and the 10.13039/501100013342NIHR Imperial Biomedical Research Centre. The views expressed are those of the authors and not necessarily those of the NIHR or the Department of Health and Social Care. M.H. is funded by 10.13039/100004440Wellcome (grant nos. 221052/Z/20/Z and 215116/Z/18/Z), the 10.13039/501100001255Lister Institute of Preventive Medicine, 10.13039/501100000272NIHR, and 10.13039/501100012295Newcastle Biomedical Research Centre. L.M.D. is supported by the European Union’s Horizon 2020 research and innovation programme under Marie Skłodowska-Curie grant agreement no. 955321. L.K. is supported by an EMBO Postdoctoral Fellowship (grant no. ALTF 120-2023) and a Royal Society Newton International Fellowship (grant no. NIF-R1-232597). J.E.P. is supported by a fellowship from the 10.13039/501100009187Medical Research Foundation (grant no. MRF-057-0003-RG-PETE-C0799). M.C.P. is a Wellcome Trust Senior Fellow in Clinical Science (grant no. 212252/Z/18/Z). C.L.C. is supported by an Auchi Clinical Research Fellowship. The authors thank Alexander Predeus for his support with the viral read analysis and Lisa Marie Milchsack and Yizhou Yu for support with the sample demultiplexing and *HLA* inference steps. We thank the anonymous peer reviewers whose input improved the paper.

## Author contributions

Conceptualization, M.B., S.A.T., M.H., M.R.C., and J.E.P. Investigation, E. Stephenson, N.B.B., M.C., A.P., E.P., T.H.M., and A.P. Methodology, J.R.F., B.J.S., and J.G. Resources, C.L.C., N.M.-T., M.P., S.M., M.W., and E. Sandhu. Formal analysis, E. Stephenson, E.M.-D., L.M.D., R.G.H.L., L.K., Z.K.T., W.M.T., and S.B. Writing – original draft, E. Stephenson, E.M.-D., L.M.D., R.G.H.L., Z.K.T., W.M.T., D.C.T., and J.E.P. Writing – review & editing, M.C.P., M.B., S.A.T., M.H., and M.R.C. Supervision, M.B., S.A.T., M.H., M.R.C., D.C.T., and J.E.P.

## Declaration of interests

S.A.T. is on the advisory board of *Cell Genomics*. L.M.D., R.G.H.L., and S.A.T. are inventors on a filed patent related to the detection and application of activated T cells. In the past 3 years, S.A.T. has received remuneration for scientific advisory board membership from Sanofi, GlaxoSmithKline, Foresite Labs, and Qiagen. S.A.T. is a co-founder and holds equity in Transition Bio and Ensocell. From January 8, 2024, S.A.T. is a part-time employee of GlaxoSmithKline.

## STAR★Methods

### Key resources table

**Table:** 

REAGENT or RESOURCE	SOURCE	IDENTIFIER
**Antibodies**		

TotalSeq™-C Human Universal Cocktail, V1.0	Biolegend	Cat# 399905

**Biological samples**		

Human blood samples	Imperial College Healthcare NHS Trust Renal and Transplant Center and its satellite dialysis units, London, United Kingdom	UK National Health Service (NHS) Health Research Authority (HRA) and Health and Care Research Wales (HCRW) Research Ethics Committee (ref. 20/WA/0123

**Chemicals, peptides, and recombinant proteins**		

Lymphoprep	STEMCELL Technologies	Cat# 18060
EasySep Dead Cell Removal	STEMCELL Technologies	Cat# 17899
Fc Receptor Blocking Solution	Biolegend	Cat# 422301

**Critical commercial assays**		

Chromium Next GEM Single Cell 5′ Kit v2	10x Genomics	Cat# 1000263
Library Construction Kit	10x Genomics	Cat# 1000190
Chromium 5′ Feature Barcode Kit	10x Genomics	Cat# 1000541
Chromium Single Cell Human TCR Amplification Kit	10x Genomics	Cat# 1000252
Chromium Single Cell Human BCR Amplification Kit	10x Genomics	Cat# 1000253
Chromium Next GEM Chip K Single Cell Kit	10x Genomics	Cat# 1000286
Dual Index Kit TT Set A	10x Genomics	Cat# 1000215
Dual Index Kit TN Set A	10x Genomics	Cat# 1000250

**Deposited data**		

Raw and analyzed data	This paper	www.covid19cellatlas.org

**Software and algorithms**		

CellRanger (v4.0)	10x Genomics	https://www.10xgenomics.com/support/software/cell-ranger/latest
SoupX	Young and Behjati^37^	https://github.com/constantAmateur/SoupX
cardelino	McCarthy et al.^38^	https://github.com/single-cell-genetics/cardelino
pysam (v0.17.0)	Heger et al.	https://github.com/niyunyun/pysam/tree/master
souporcell (v2.0)	Heaton et al.^39^	https://github.com/wheaton5/souporcell
leidenalg (v0.8.9)	Traag et al.^40^	https://github.com/vtraag/leidenalg
Seurat (v4.1.1)	Butler et al.^41^	https://satijalab.org/seurat/articles/install_v5.html
harmony (v1.0)	Korsunsky et al.^42^	https://github.com/immunogenomics/harmony
harmonypy (v0.0.6)	Slowikowski et al.	https://github.com/slowkow/harmonypy
STARsolo (STAR release 2.7.10a_alpha)	Dobin et al.	https://github.com/alexdobin/STAR/tree/master
MSigDB (v7.5)	Broad Institute, Inc., MIT	https://www.gsea-msigdb.org/gsea/msigdb
scuttle (v1.9.0)	McCarthy et al.^38^	https://www.bioconductor.org/packages/release/bioc/html/scuttle.html
FactoMineR (v2.4)	Le et al.^43^	https://cran.r-project.org/web/packages/FactoMineR/index.html
MiloR (v.0.99.0)	Dann et al.^29^	https://marionilab.github.io/miloR/index.html
cellranger-vdj (v.6.0.0)	10x Genomics	https://www.10xgenomics.com/support/software/cell-ranger/latest/tutorials/cr-tutorial-vdj
scirpy (v1.10.1)	Sturm et al.^44^	https://scirpy.scverse.org/en/latest/index.html
dandelion (v.0.2.4)	Suo et al.^45^	www.github.com/zktuong/dandelion
celltypist (v1.2.0)	Dominguez Conde et al.^11^	https://www.celltypist.org/
cell2tcr (v0.1)	Lindeboom et al.^25^	https://github.com/Teichlab/cell2tcr
python (v3.10.2)	van Rossum et al.	https://www.python.org/downloads/
pandas (v1.4.2)	NumFOCUS	https://pandas.pydata.org/
numpy (v1.21.6)	Harris et al.	https://numpy.org/
scanpy (v1.9.1)	Wolf et al.^46^	https://scanpy.readthedocs.io/en/latest/
matplotlib (v3.5.2)	Hunter	https://matplotlib.org/
seaborn (v0.11.2)	Waskom et al.	https://seaborn.pydata.org/
scipy (v1.8.1)	Virtanen et al.	https://scipy.org/
statannotations (v0.5.0)	Charlier et al.	https://github.com/trevismd/statannotations
sinto (v0.10.1)	Stuart et al.	https://timoast.github.io/sinto/
samtools (v1.19.2)	Danecek et al.	https://github.com/samtools/samtools
arcas-hla (commit 9fa54a212d134b0d9894d1fc19ec1bdc6f62eb55)	Orenbuch et al.	https://github.com/RabadanLab/arcasHLA
scVI (v.0.19.0)	Lopez et al.^47^	https://scvi-tools.org/
gseapy (v.0.10.8)	Fang et al.	https://github.com/zqfang/GSEApy

### Experimental model and study participant details

#### Ethical approval

All participants (patients and controls) were recruited from the Imperial College Healthcare NHS Trust Renal and Transplant Center and its satellite dialysis units, London, United Kingdom, and provided written informed consent prior to participation. Study ethics were reviewed by the UK National Health Service (NHS) Health Research Authority (HRA) and Health and Care Research Wales (HCRW) Research Ethics Committee (ref. 20/WA/0123: The impact of COVID-19 on patients with renal disease and immunosuppressed patients). Ethical approval was given.

#### Patient cohorts

We recruited two cohorts of ESKD patients with COVID-19 (Table S13). All patients were on haemodialysis prior to acquiring COVID-19. Sample and patient numbers reported here refer to numbers used in the reported analyses i.e., after exclusions due to quality control (QC) failures. The first cohort (‘2020/Wave 1’) was recruited during the initial phase of the COVID-19 pandemic (April-May 2020). 61 serial blood samples collected during acute COVID-19 infection from 21 ESKD patients were available for analysis. Three samples were collected for 19 of these patients; two samples were collected for the other two individuals. We also contemporaneously recruited non-infected ESKD patients on haemodialysis to provide a control group (*n* = 37).

The second cohort (‘2021/Wave 2’) were recruited during the resurgence of COVID-19 cases in January-March 2021. This cohort, which consisted of 16 ESKD patients with COVID-19, had all been recruited as part of the COVID-19 negative control group during the 2020 Wave, and so a pre-infection sample collected in April/May 2020 (8–9 months preceding infection) was also available for 13 patients (for 3 patients samples were unavailable due to insufficient cells and/or QC failures). For patients with COVID-19 in Wave 2, samples were systematically acquired at regular intervals (median 5 samples per patient, collected every 2–3 days over the course of the acute infection). Additionally, for 10 of these 16 patients, we acquired convalescent samples approximately 2 months following the acute COVID-19 episode. Three individuals in this cohort had received one dose of a COVID-19 vaccine shortly before COVID-19 diagnosis (maximum time 5 days from vaccination to illness i.e., before any protective effect of vaccination would be expected to occur).

For CITE-seq and comparisons of COVID-19 positive versus negative samples, the 2021/Wave 2 COVID-19 positive samples were processed and analyzed with their corresponding COVID-19 negative (pre-infection samples) from 2020. These 16 COVID-19 negative samples were excluded from the 2020/Wave 1 control group to avoid re-use of the same control samples across analyses. As a result, the Wave 1 analysis used 24 COVID-19 negative control samples.

#### Clinical severity scores

Severity scoring was performed based on WHO classifications (WHO clinical management of COVID-19: Interim guidance 27 May 2020) adapted for clinical data available from electronic medical records. ‘Mild’ was defined as COVID-19 symptoms but no evidence of pneumonia and no hypoxia. ‘Moderate’ was defined as symptoms of pneumonia or hypoxia with oxygen saturation (SaO2) greater than 92% on air, or an oxygen requirement no greater than 4 L/min ‘Severe’ was defined as SaO2 less than 92% on air, or respiratory rate more than 30 per minute, or oxygen requirement more than 4 L/min ‘Critical’ was defined as organ dysfunction or shock or need for high dependency or intensive care support (i.e., the need for non-invasive ventilation or intubation). Severity scores were charted throughout a patient’s illness. We defined the overall severity/clinical course for each patient as the peak severity score that occurred during the patient’s illness. Contemporaneous severity scores (i.e., severity score at the time of the blood sample) were used for the differential gene expression analyses used to identify gene expression associated with COVID-19 severity. Clinical course (peak illness severity) was used to define the severity strata for modeling the longitudinal gene expression profiles.

### Method details

#### PBMC isolation

Peripheral blood mononuclear cells (PBMCs) were obtained by density gradient centrifugation using Lymphoprep (STEMCELL Technologies, Canada). Approximately 20 mL of blood were diluted 1× with phosphate buffered saline (PBS) with addition of 2% fetal bovine serum (FBS) and layered on top of 15 mL of Lymphoprep solution. The samples were then centrifuged at 800 g for 20 min at room temperature without break. PBMCs were collected from the interface and washed twice with PBS/2%FBS. PBMCs were cryopreserved in 1 mL freezing medium (FBS 10% DMSO) and stored in liquid nitrogen. PBMC isolation for all samples was performed at Imperial College London, UK.

#### PBMC processing and CITEseq

##### Samples collected during 2020 wave

Frozen PBMCs were thawed by adding a small volume of ice-cold PBS to PBMC samples and transferred to a falcon tube containing 35 mL of ice-cold PBS. Samples were then centrifuged and counted. Dead cells were removed using the EasySep Dead Cell Removal kit (Stem Cell Technologies) according to the manufacturer’s protocol. Cells were then counted again and 40,000 cells from each sample were pooled together in batches of seven with the aim for each pool to contain ∼300,000 cells, ensuring each pool had a different combination of genotypes for simple demultiplexing. Pooled cells were then stained with Fc Receptor Blocking Solution (Biolegend) and then with TotalSeq-C Human Universal Cocktail V1.0 (Biolegend) according to the manufacturer. Cells were then washed once with PBS and then counted. Each pool was loaded across two channels of a Chromium Chip (10x Genomics), using Single Cell 5′ V2 kits, to achieve a recovery of 10,000 cells per sample. The sample processing described above was performed at the University of Newcastle, UK.

##### Samples collected during 2021 wave and pre-infection samples from 2020

Frozen PBMCs were thawed at 37°C until a small ice crystal remained. Samples were then transferred to another tube and ten times the volume of pre-warmed RF-10 media (RPMI (Sigma) supplemented with 10% (v/v) fetal calf serum (Life technologies), 100U/ml Penicillin (Sigma), 100 μg/mL Streptomycin (Sigma) and 1% (v/v) L-Glutamine) was added dropwise. Cells were then centrifuged and counted. Dead cells were removed using the EasySep Dead Cell Removal kit (Stem Cell Technologies) according to the manufacturer’s protocol. Cells were then counted again and 250,000 cells from each sample were pooled together in batches of four using a leave-one-out strategy for simple demultiplexing. Pooled cells were then stained with Fc Receptor Blocking Solution (Biolegend) and then with TotalSeq-C Human Universal Cocktail V1.0 (Biolegend) according to the manufacturer. Cells were then washed three times with Flow Buffer (Dulbecco’s phosphate buffered saline (PBS)(Sigma) supplemented with 2% (v/v) FCS and 2mM EDTA (Sigma)) and then counted. Each pool was loaded across two channels of a Chromium Chip (10x Genomics), using Single Cell 5′ V2 kits, to achieve a recovery of 10,000 cells per sample. The sample processing described above was performed at the University of Cambridge, UK.

#### Library preparation and sequencing

Gene expression, cell surface protein, TCR and BCR libraries were generated according to the manufacturer’s protocols. All libraries were sequenced using a NovaSeq 6000 at the Wellcome Sanger Institute, UK, to achieve a minimum of 20,000 reads per cell for gene expression libraries and 5,000 reads for cell surface protein, TCR and BCR libraries.

#### Initial data processing and QC

We jointly aligned the antibody-derived tags (ADT) and gene expression libraries from CITE-seq experiments using *CellRanger 4.0*, using the reference 10X Genomics provided with the release of *CellRanger 3.0*, and the ADT barcode reference provided by the supplier. Single cell TCR and BCR sequencing data was aligned using *CellRanger 4.0* using the GRCh38 VDJ reference provided by 10X Genomics. We used *Seurat* V4.1.0^41^ to import gene expression and ADT counts. Low quality cells were excluded by removing droplets with either fewer than 1000 RNA UMIs, or fewer than 200 RNA features detected, or with more than 10% of their RNA UMIs mapping to mitochondrial genes. *SoupX*^37^ was used to remove signals from ambient RNA and background antibody staining. SoupX parameters ‘soupQuantile’ and ‘tfidfMin’ were set to 0.25 and 0.2, respectively, and lowered by decrements of 0.05 until the contamination fraction was calculated using the ‘autoEstCont’ function. Corrected gene expression and ADT counts were then scaled to 10000 UMIs per cell and log1p transformed.

#### Sample demultiplexing

We used *souporcell v2.0*^39^ to perform genotype-based demultiplexing of pooled PBMC libraries to assign donor identifiers to each single cell transcriptome. To ensure high reproducibility of the genotype-decomposition, we merged the sequencing data from each set of replicates of the same donor pool prior to *souporcell* analysis. We used *pysam v0.17.0* to amend cell barcodes with original library identifiers and to merge bam files. Using the merged bam files, we ran *souporcell* using the provided set of common variants, with remapping disabled and with the appropriate number of expected genotypes. To assign a donor identifier to each *souporcell* genotype cluster we leveraged the pooling strategy of donors per library which was designed in such a way that every donor was present in a unique combination of pools. We used the *cardelino* R package^38^ to import genotypes and perform pairwise comparisons of all identified *souporcell* genotype clusters, to identify highly similar genotype clusters in different pools that likely originated from the same donor, which was then given a donor label based on the combination of pools in which the genotype was detected. Genotypes that were not resolvable due to missing or low-quality data, were excluded from downstream analyses.

We detected a total of 1,337,786 cells with at least 200 genes quantified. We next applied stringent filtering on cell quality to remove cells with more than 10% mitochondrial reads and cells with less than 1000 UMIs quantified. In addition, we only kept cells with a genotype/patient id assignment using souporcell, and that did not cluster in doublet enriched leiden clusters during the manual annotation process. Samples from two individuals were observed to not integrate well and they were subsequently identified to be samples from patients with benign chronic lymphocytic leukemia and were removed from all downstream analyses (Table S1). This resulted in a dataset of 580,040 high-quality cells from 61 patients and 187 samples that were used for the reported analyses.

#### Broad cell annotation

We first split the whole dataset into three compartments: myeloid and non-immune haematopoietic, T and NK cells, and B cells. To do this, we devised a custom *CellTypist**^8^* model consisting of publicly available COVID-19 datasets^12^^,^^13^ from PBMC samples. The predicted cell labels were then used to broadly split the data into the three compartments which were subsequently processed as described below.

#### Annotation—Myeloid and non-immune

Annotation of myeloid and progenitor compartment was performed using *scanpy*^46^ (v1.8.2). The dataset was initially normalized, and log transformed, and then filtered for highly variable genes (*scanpy.pp.highly_variable_genes; min_mean = 0.0125, max_mean = 3, min_disp = 0.5*) and scaled (*scanpy.pp.scale, max_value = 10*). Dimensionality reduction was performed using principal component analysis (PCA; *scanpy.tl.pca*), and integration was done using *harmony*^42^ (*harmonypy*, v0.0.6). Clustering was performed using the Leiden^40^ algorithm (*leidenalg,* v0.8.9). The marker genes for each cluster were examined using the function ‘*scanpy.tl.rank_genes_groups*’ and each cluster was manually annotated.

#### Annotation—T and NK cell compartment

The T and NK cell compartment quality control and annotation was performed using *scanpy*^46^(v1.9.8). The dataset was initially normalized, and log transformed, and then filtered for highly variable genes (*scanpy.pp.highly_variable_genes; min_mean = 0.0125, max_mean = 3, min_disp = 0.5*). Unwanted sources of variation in the form of total read count and percentage of mitochondrial genes were regressed out (using the *scanpy.pp.regress* function). The gene expression data was then scaled (*scanpy.pp.scale, max_value = 10*) and dimensionality reduction was performed using principal component analysis (PCA; *scanpy.tl.pca*). The first 40 principal components were used to compute a nearest neighbors distance matrix (*scanpy.pp.neighbors*), which was subsequently embedded using Uniform Manifold Approximation and Projection (UMAP; *scanpy.tl.umap*). Cell clustering was performed using the Leiden algorithm (*scanpy.tl.leiden*). The resulting clusters were manually annotated using canonical marker genes through an iterative process of re-clustering, annotation, and re-clustering. CITE-seq marker proteins CD45RA and CD45RO were used to distinguish naive and T EMRA from other memory T cell subsets, respectively. All other markers used for annotations were based on mRNA expression data.

#### Annotation—B cell

The B cell compartment was integrated using *scVI* (v.0.19.0)^47^ with sequencing samples (‘*orig.ident*’) as the batch key and raw count data as input. Percentage mitochondrial content and total counts were provided as continuous variables to the *scVI* model. Feature selection prior to setting up the scVI model was performed as per standard procedures in *scanpy.pp.highly_variable_genes* with *min_mean = 0.0125, max_mean = 3, min_disp = 0.5*, using the log transformed normalized expression data (normalized to 10,000 counts per cell). BCR V(D)J genes were also removed from the highly variable features. Expression of canonical B cell and ASC marker genes and non-B cell markers were then assessed to manually determine potential multiplets, over iterative rounds of sub-clustering. The annotations were also assessed against a publicly available bulk RNAseq gene set of major PBMC cell types.^48^ In addition, the single-cell scores computed after enrichment of the bulk RNA-seq signatures were fitted into a two-component Gaussian mixture model (*max iter = 1000, covariance_type = 'full'*) which distinguished ASCs from non-ASC B cell clusters. Subsequent sub-clustering and annotations were performed on the ASCs and non-ASCs separately. To annotate the non-ASC cell clusters, mRNA and surface molecule expression for select targets (CITE-seq; CD11C and CD27), along with the Monaco et al.^48^ peripheral blood B cell signatures. Isotype usage was checked using the single-cell and BCR-seq information and used to manually update the cell type annotations, ensuring that naive B cells, non-switched memory B cells and IgM ASCs are only associated with IgM and/or IgD while switched memory B cells and IgA/IgG ASCs are only associated with IgG/IgA isotypes. Other antibody isotypes expressing ASCs (IgD/IgE) are labeled as ‘*B_ASC_others*’.

#### Mapping viral reads

To detect viral RNA, we combined the Human GRCh38 reference genome (GENCODE v32) with 21 viral genomes including SARS-CoV-2 (NC_045512.2) for RNA-seq alignment, as previously described.^13^ All samples were re-mapped to the extended reference genome using STARsolo from STAR release 2.7.10a_alpha. Viral reads were only considered for cells that had passed the previously described QC thresholds. To determine the distribution of viral reads across immune cells, total viral read counts were summed across all samples and grouped by broad cell type and viral species.

#### Cell-type composition analysis

The cell type abundances per sample were modeled using a generalised linear mixed model using a poisson outcome as described in Yoshida et al*.*^13^ We fitted log_2_ transformed age, and random effect terms on biological sex and ethnicity, to account for collinearity with features of interest. We also fitted a random effect term on the donor identifier to account for donor-to-donor variation but captured the paired effects between longitudinal samples from the same donor. To perform longitudinal analyses, we modeled weeks since onset of disease (onset of symptoms or positive test, whichever came first) as categorical features, and scaled the conditional distribution of fold change estimates to the COVID-19 negative samples that were available, and the COVID-19 negative standard deviation was multiplied by the standard deviation of each other timepoint factor level to account for the increased variance that is introduced by scaling. The same modeling framework was used to assess cell type abundance changes with disease severity, with fold change estimated using mild/moderate COVID-19 positive samples as the reference group.

#### Genetic principal component analysis

To overcome missing and potentially unreliable self-reported ethnicity data for some donors, we used PCA on genotyping data to infer genetic ancestry. We took the *souporcell* cluster genotypes of all donors and converted them into a numerical matrix to perform PCA on using *FactoMineR V2.4*.^43^ We then mapped self-reported ethnicity onto the genetic PCA results. This revealed that principal component 1 separated individuals with self-reported ethnicities indicating African ancestry from individuals with other ethnicities, while principal component 2 separated individuals of self-reported Asian ancestry from those with self-reported European ancestry. To adjust for the potential confounding effects of ethnicity (since ethnicity is associated with higher risk of severe and fatal COVID-19), we included these 2 principal components (continuous variables) as covariates in all linear mixed models (Table S1, Figure S7).

#### Differential gene expression analysis

Single-cell data was separated by cell type. Raw count data were aggregated by sample using the function ‘*scuttle::aggregrateAcrossCells*’; only samples with more than 10 cells were taken forward for downstream analysis. Using an edgeR workflow, pseudo-bulked data was converted into a *DGEList* object using *‘edgeR::DGEList’* and low expression genes were removed (‘*filterByExpr()’, min.count = 3, min.total.count = 5*). Normalisation factors were calculated and the negative binomial dispersions for each gene estimated using *‘edgeR::calcNormFactors’* and *‘edgeR::estimateDisp’* respectively. To account for serial sampling in our data (i.e., non-independence of samples), mixed effects negative binomial models were fit using *‘lme4::glmer’*. Log transformed effective library sizes (library size multiplied by *edgeR* normalisation factors) and estimated dispersions were supplied to the model via the offset argument. Any genes with *lme4* convergence warnings were removed.

We performed differential gene expression analysis comparing COVID-19 positive to COVID-19 negative samples (all from patients with ESKD) separately for each cohort using the following model:

Expression∼case_control+sex+age_scaled+genetic_PC1+genetic_PC2+(1|individual_id)

Similarly, to test for gene expression changes associated with COVID-19 severity at the time of blood sampling, we perform differential gene expression analysis comparing samples taken at the time of severe/critical COVID-19 to samples taken at the time of mild/moderate COVID-19 using the following model, where center indicates the site of library preparation (2020 Cohort/Wave 1: Newcastle, 2021 Cohort/Wave 2: Cambridge).

Expression∼WHO_temp_severity_group+sex+genetic_PC1+genetic_PC2+age_scaled+centre+(1|individual_id)

#### Gene set enrichment analysis

Gene set enrichment analysis (GSEA) was performed on ordered ranked gene lists from the differential gene expression analyses comparing i) COVID-19 positive and COVID-19 negative samples and ii) severe/critical COVID-19 positive samples and mild/moderate COVID-19 negative samples. GSEA was conducted using the ‘*clusterProfiler::GSEA*’ function in R, with genesets defined by ‘*msigdbr::msigdbr(species = "human", category = "C2")*’. *p*-values were adjusted using Benjamini-Hochberg to account for multiple testing. Where significant enrichment of pathways was identified within a cell type, we then used a more granular cell type annotation to delineate the source of the signal.

#### Differential abundance during glucocorticoid treatment

We examined the effect of glucocorticoid treatment on the cell abundance using the MiloR package (v.0.99.0).^29^ The monocyte population was subsetted to include only the samples from COVID-19 positive patients who received the steroid treatment during Wave 2 (2021) of COVID-19. A KNN graph was constructed using the function ‘*buildGraph*’ (*k = 30, d = 30*) and the cells were assigned to the neighbourhoods on the KNN graph using the function ‘*makeNhoods*’ (*prop = 0.1, k = 30, d = 30*). The number of cells belonging to each sample in each neighborhood was counted using the function ‘*countCells*’. We included ‘*time_from_infection*’ in the design to account for the length of disease. SpatialFDR <0.1 was used as a cut off point for significant enrichment/depletion.

#### Longitudinal transcriptomic analysis

We defined time from infection as the time from first symptoms, or time from first positive nasal swab if the latter preceded symptoms (since some cases of COVID-19 were identified by screening procedures in place for patients attending haemodialysis).

For longitudinal analysis of enrichment of MSigDB (v7.5) Hallmark, KEGG and Reactome genesets,^49^ the single-cell data was separated to each cell type and the raw count data was aggregated by sample using ‘*scuttle::aggregrateAcrossCells*’ (v1.9.0). Only samples with more than 10 cells were used for downstream analysis. The pseudo-bulked data was then log transformed and normalized using ‘*scuttle::logNormCounts*’ and converted to a module score using ‘*Seurat::AddModuleScore*’. The module scores were then tested for differential enrichment over time according to severity strata, using a general linear mixed-effect model with ‘*lme4:lmer*’ using the following formula:

geneset∼sex+age_scaled+(1|individual_id)+centre+

geneticPC1+geneticPC2+

splines::bs(time_from_infection,degree=2)∗severity

“Severity” here represents overall clinical course, defined by peak illness severity, binarised into either severe/critical or mild/moderate. The estimated marginal means for the first 21 days from infection for the relevant genesets were computed using ‘emmeans:emmeans’ with ‘*time_from_infection*’ by ‘*grouped_severity*’. *p* values were adjusted using the Benjamini-Hochberg procedure.^50^

#### BCR and TCR data processing

Single-cell BCR and TCR data were initially processed with cellranger-vdj (v.6.0.0). Single cell TCR data was then converted into a cell by TCR format using scirpy v1.10.1.^44^ BCR contigs contained in all_contigs.fasta and all_contig_annotations.csv were then processed further using *dandelion*^45^ singularity container (v.0.2.4) (https://www.github.com/zktuong/dandelion). BCRs were then matched to cell barcodes with *dandelion*.

#### TCR analysis

After quality control, we recovered 197,330 T cells with fully resolved T cell receptors from 61 donors and across 187 samples. We identified 127,670 unique TCR clones, defined by a unique combination of CDR3a, TRAV, TRAJ, CDR3B, TRBV, TRBJ and donor, at the amino acid level. Of these, 93,960 came from COVID-19 positive ESKD patients and thus could be analyzed longitudinally over the course of infection. A total of 3,727 clones (4%) were captured at two or more time points during infection. We further excluded all clones present in pre-pandemic samples for analysis related to COVID-19, as these could not have expanded in response to SARS-CoV-2, and finally obtained 3,137 clones (3.3%) for longitudinal analysis. Clonal frequency within a sample was calculated as the total number of clone copies per sample over the total number of T cells within the sample. To determine expansion, only clones that were sampled at two time points or more within the 0 to 30 days after a positive PCR nasal swab, and that were absent in the pre-COVID-19 samples, were used. An expansion was noted if the highest clone frequency measured before a specific day since positive swab (cutoff) was lower than the lowest frequency measured after that day. If the clone was not sampled either before or after the cutoff, the respective frequency was set to 0. The cutoff at day 10 was selected as being in agreement with timing of an adaptive immune response. For the more stringent definition of expansion as determined by a dual cutoff, the clone frequency had to show an increase at the first cutoff and a further increase at the second cutoff. This allowed the capture of a steeper increase of clonal frequency over time, at the cost of considering fewer total clones.

SARS-CoV-2 specific TCR-epitope pairs were queried from VDJDB. Samples from before the pandemic were used to establish a baseline of matches with the database. While a single-chain match with the database only indicates a putatively binding TCR, quantifying significant differences in these numbers across T cell populations gives insight into antigen specificity. Matches with the database were quantified for expanding and non-expanding clones using bar charts, where the error bars show variation across individual COVID-19 patients, and significance was determined with a two-sided Mann-Whitney test. To determine which clones were expanding the most, expansion was determined as the mean clonal frequency after the cutoff day divided by the mean clonal frequency before and sorted in descending order.

Activated T cells were identified by applying the automatic cell type classifier Celltypist (1.2.0, model = COVID-19_HumanChallenge_Blood) and sub-setting to activated T cells. Cell2TCR (0.1) was used on the clones that showed expansion according to the above definition using days 2 and 10 as dual cutoffs, and to generate TCR motifs, while excluding TCR sequences of MAI T cells.

TCR analyses were carried out in Python (3.10.2) using pandas (1.4.2), numpy (1.21.6) and scanpy (1.9.1), and visualised with matplotlib (3.5.2) and seaborn (0.11.2), in particular seaborn’s lineplot to show clonal frequency evolution. Statistical tests were carried out using the scipy.stats module (1.8.1) and plotted with statannotations (0.5.0). The regression line and R2 value were determined with the seaborn’s regplot function.

#### HLA inference

For inference of HLA types from the raw single cell sequencing reads, data from pooled samples were subset to chromosome 6 reads, split and merged by donor with packages sinto (0.10.1) and samtools (1.19.2). HLA genes were then typed using arcas-hla (built directly from source https://github.com/RabadanLab/arcasHLA at commit 9fa54a212d134b0d9894d1fc19ec1bdc6f62eb55), yielding six-digit HLA type predictions for all donors. HLA types are in Table S10.

#### Integration of ESKD and non-ESKD cohorts

To test whether there were differences in COVID-19 associated transcriptomic changes in ESKD versus non-ESKD, we integrated our dataset with two non-ESKD COVID-19 single cell transcriptomic datasets^12^^,^^34^, obtained from https://zenodo.org/record/6120249#.Y49penbP1aQ. Datasets were processed with the single cell analysis Python workflow Scanpy.^46^ For the Stephenson et al. dataset, cells from healthy individuals who had received lipopolysaccharide challenge and from individuals hospitalised with non-COVID-19 comparator illnesses were excluded, while for the COMBAT study dataset, cells from patients with bacterial sepsis or influenza were removed, to retain only COVID-19 and healthy control samples. Each dataset was individually filtered, with every cell required to express at least 200 and at most 3,500 genes, of which less than 10% mitochondrial counts, with all other parameters kept at default values. Genes expressed in fewer than 5 cells were removed. For integration, we took the intersection of genes across all three datasets and subset to those genes before computing 6000 highly variable genes. The Celltypist model ‘COVID-19_HumanChallenge_Blood’^11^ was applied on all cells to obtain common cell type annotations.

A probabilistic scVI^47^ model with two hidden layers, 128 hidden nodes per layer, 20-dimensional latent space, 6000 highly variable genes and negative binomially distributed gene likelihood was trained on the data for 40 epochs, with all other parameters kept at default values. All cells were mapped to a shared latent space. A visualization of the embedding was obtained using UMAP. The scVI model was used to perform random sampling in the gene expression space, which can also yield differentially expressed genes (DEGs). For this, cells were indexed by conditions of interest (such as cell type and ESKD status), gene expression level distributions and effect sizes per condition estimated, and posterior expectations computed at a False Discovery Proportion below a significance level of 0.01 to determine which genes were differentially expressed between conditions, using the built-in scVI.model.differential_expression function. Only comparisons for which each condition comprised at least 50 cells were carried out. Results of DEG analysis are in Table S11.

Over-representation analysis of Gene Ontology (GO) pathways contained in the ‘GO_Biological_Process_2021’ set by upregulated (LFC>0) and downregulated (LFC<0) genes were performed using the enrichr module in gseapy (v.0.10.8) separately. A Benjamini-Hochberg adjusted *p*-value <0.05 was used as a cut-off value for significant terms.

The TGF-beta gene signatures for all cells were scored using Scanpy function scanpy.tl.score_genes^46^ and aggregated by ESKD status. The score is computed per-cell as the average expression of the target genes subtracted with the average expression of a reference set of genes. The reference set is randomly sampled from the data for each binned expression value. We also scored the three genes *TGFB1*, *TGFB2*, and *TGFB3* to investigate whether these were driving the signal (scores in Table S12).

#### Mendelian randomization

We performed two-sample MR to test whether PLAC8 plays a causal role in COVID-19 severity. Analysis was performed using the TwoSampleMR package. We used eQTLs for *PLAC8* gene expression as genetic instruments. Whole blood eQTL data from eQTLGen phase I (*N* = 31,684) (https://molgenis26.gcc.rug.nl/downloads/eqtlgen/cis-eqtl/2019-12-11-cis-eQTLsFDR0.05-ProbeLevel-CohortInfoRemoved-BonferroniAdded.txt.gz) were filtered to remove variants in linkage disequilibrium (r^2^ < 0.001). This procedure resulted in rs10021035 and rs76694191 as instrumental variables (IVs). For the outcome data, we used the GWAS of very severe respiratory confirmed COVID-19 versus population by the COVID-19 Host Genetics Initiative (Release 7, European ancestry excluding 23andME, N cases = 13,769). We used the inverse-weighted variance method as the primary analysis. Sensitivity analyses included assessing single SNP Wald ratio results. Since there were fewer than three IVs we were unable to perform formal tests for heterogeneity or pleiotropy of instruments.

#### Integration of Olink plasma proteomics

A subset of Wave 1/2020 Cohort (45 individuals, 85 samples) had plasma proteomic measurements from 5 Olink Target 96 panels: ‘cardiometabolic’, ‘cardiovascular 2’, ‘cardiovascular 3’, ‘inflammation’ and ‘immune response’. The Olink proteomics data for these samples has previously been described.^22^

## Supplemental information

Document S1. Figures S1–S7

Table S1. Sample metadata, related to STAR Methods

Table S2. List of antibodies included in the TotalSeq panel, related to STAR Methods

Table S3. Differential cell abundance analysis comparing COVID-19^+^ samples with COVID-19^−^ samples, stratified by week 1 or week 2 of illness, related to Figures 2A–2J

Table S4. Differential gene expression analysis comparing COVID-19^+^ with COVID-19^−^ samples within each cell type, related to STAR Methods

Table S5. Gene set enrichment analysis of differentially expressed genes in COVID-19^+^ versus COVID-19^−^ samples by cell type, related to Figure 2K

Table S6. Differential cell abundance analysis comparing severe/critical COVID-19^+^ samples to mild/moderate COVID-19^+^ samples, related to Figure 3A

Table S7. Differentially expressed genes for each cell type from comparison of severe/critical COVID-19^+^ samples to mild/moderate COVID-19^+^ samples, related to STAR Methods

Table S8. Gene set enrichment pathway analysis for each cell type based on DEGs from comparison of severe/critical COVID-19^+^ samples to mild/moderate COVID-19^+^ samples, related to Figure 3B

Table S9. Pathways showing significant time × severity interactions, related to Figure 4B

Table S10. Inferred HLA types for all donors, related to STAR Methods

Table S11. DEGs from scVI model for broad cell compartments, related to STAR Methods

Table S12. TGF-β signature score and TGF-β1-TGF-β2-TGF-β3 scores, related to Figures 6D–6G

Table S13. Patient variables stratified by cohort, related to STAR Methods

Document S2. Article plus supplemental information
