# Supplementary material for: Temporal multi-omics analysis of COVID-19 in end-stage kidney disease
Source: Cell Genom. 2025 Jun 17;5(8):100918. doi: 10.1016/j.xgen.2025.100918 (PMC12366655; doi:10.1016/j.xgen.2025.100918)
Supplement: Document S1. Figures S1–S7 [file mmc1.pdf]

Cell Genomics, Volume *5*

Supplemental information

Temporal multi-omics analysis

of COVID-19 in end-stage kidney disease

Emily Stephenson, Erin Macdonald-Dunlop, Lisa M. Dratva, Rik G.H. Lindeboom, Zewen Kelvin Tuong, Win Min Tun, Lorenz Kretschmer, Norzawani B. Buang, Stephane Ballereau, Mia Cabantaus, Ana Peñalver, Elena Prigmore, John R. Ferdinand, Benjamin J. Stewart, Jack Gisby, Talat H. Malik, Candice L. Clarke, Nicholas Medjeral-Thomas, Maria Prendecki, Stephen McAdoo, Anais Portet, Michelle Willicombe, Eleanor Sandhu, Matthew C. Pickering, Marina Botto, Sarah A. Teichmann, Muzlifah Haniffa, Menna R. Clatworthy, David C. Thomas, and James E. Peters

Figure S1.

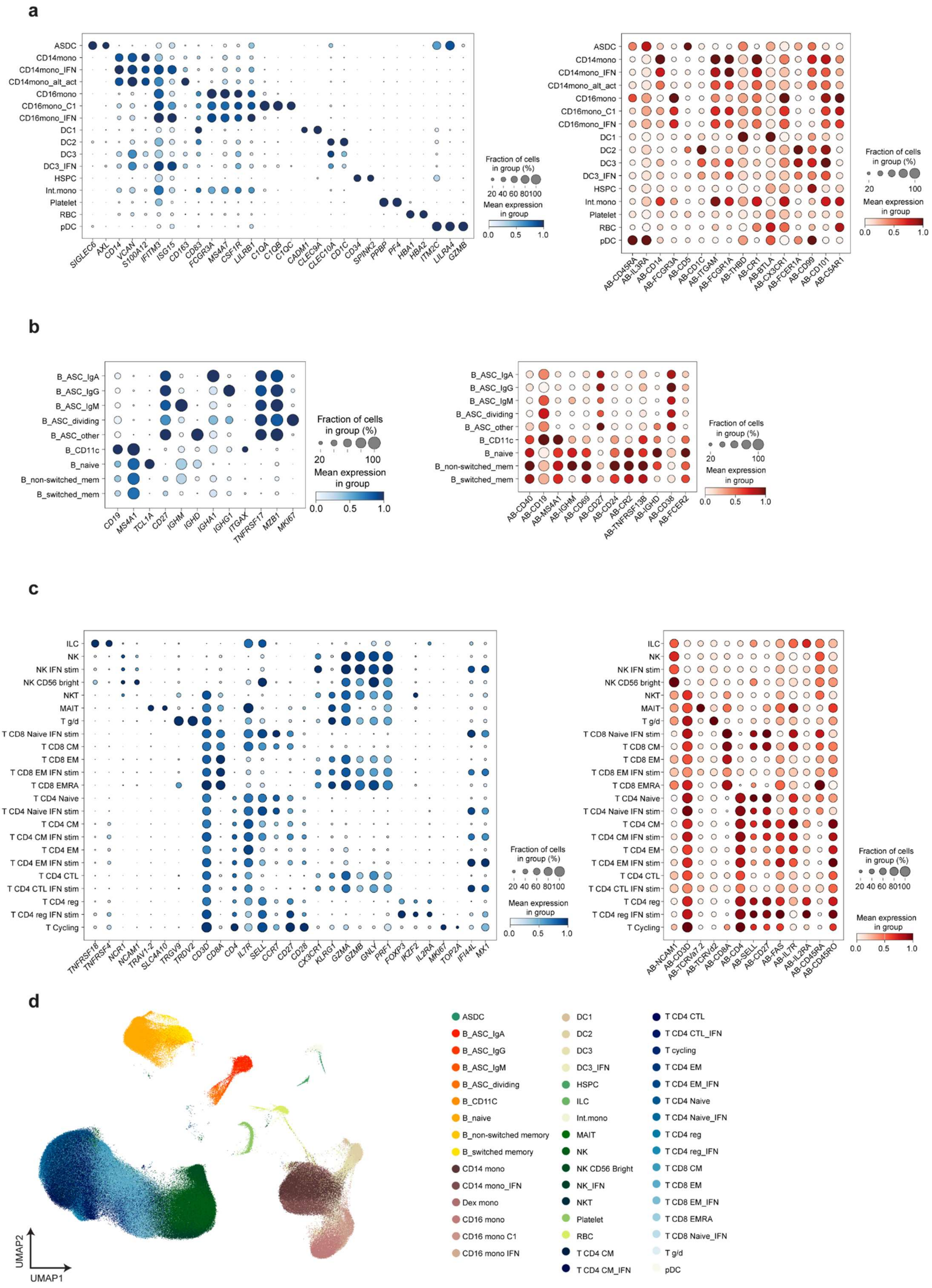
a
ASDC
CD14mono
CD14mono_IFN
CD14mono_alt_act
CD16mono
CD16mono_C1
CD16mono_IFN
DC1
DC2
DC3
DC3_IFN
HSPC
Int.mono
Platelet
RBC
pDC
SIGLEC6 AXL CD14 VCAN S100A12 IFITM3 ISG15 CD163 CD83 FCGR3A MS4A7 CSF1R LILRB1 C1QA C1QB C1QC CADM1 CLEC9A CLEC10A CD1C CD34 SPINK2 PPBP PF4 HBA1 HBA2 ITM2C LILRA4 GZMB
Fraction of cells in group (%)
20 40 60 80 100
Mean expression in group
0.0 0.5 1.0
AB-CD45RA AB-IL3RA AB-CD14 AB-FCGR3A AB-CD5 AB-CD1C AB-ITGAM AB-FCGR1A AB-THBD AB-CR1 AB-BTLA AB-CX3CR1 AB-FCER1A AB-CD99 AB-CD101 AB-C5AR1
Fraction of cells in group (%)
20 100
Mean expression in group
0.0 0.5 1.0
b
B_ASC_IgA
B_ASC_IgG
B_ASC_IgM
B_ASC_dividing
B_ASC_other
B_CD11c
B_naive
B_non-switched_mem
B_switched_mem
CD19 MS4A1 TCL1A CD27 IGHM IGHD IGHA1 IGHG1 ITGAX TNFRSF17 MZB1 MKI67
Fraction of cells in group (%)
20 100
Mean expression in group
0.0 0.5 1.0
AB-CD40 AB-CD19 AB-MS4A1 AB-IGHM AB-CD69 AB-CD27 AB-CD24 AB-CR2 AB-TNFRSF13B AB-IGHD AB-CD38 AB-FCER2
Fraction of cells in group (%)
20 100
Mean expression in group
0.0 0.5 1.0
c
ILC
NK
NK IFN stim
NK CD56 bright
NKT
MAIT
T g/d
T CD8 Naive IFN stim
T CD8 CM
T CD8 EM
T CD8 EM IFN stim
T CD8 EMRA
T CD4 Naive
T CD4 Naive IFN stim
T CD4 CM
T CD4 CM IFN stim
T CD4 EM
T CD4 EM IFN stim
T CD4 CTL
T CD4 CTL IFN stim
T CD4 reg
T CD4 reg IFN stim
T Cycling
TNFRSF18 TNFRSF4 NCR1 NCAM1 TRAV1-2 SLC4A10 TRGV9 TRDV2 CD3D CD8A CD4 IL7R SELL CCR7 CD27 CD28 CX3CR1 KLRG1 GZMA GZMB GNLY PRF1 FOXP3 IKZF2 IL2RA MKI67 TOP2A IFI44L MX1
Fraction of cells in group (%)
20 40 60 80 100
Mean expression in group
0.0 0.5 1.0
AB-NCAM1 AB-CD3D AB-TCRVa7.2 AB-TCRVd2 AB-CD8A AB-CD4 AB-SELL AB-CD27 AB-FAS AB-IL7R AB-IL2RA AB-CD45RA AB-CD45RO
Fraction of cells in group (%)
20 40 60 80 100
Mean expression in group
0.0 0.5 1.0
d
UMAP2
UMAP1
ASDC
B_ASC_IgA
B_ASC_IgG
B_ASC_IgM
B_ASC_dividing
B_CD11C
B_naive
B_non-switched memory
B_switched memory
CD14 mono
CD14 mono_IFN
Dex mono
CD16 mono
CD16 mono C1
CD16 mono IFN
DC1
DC2
DC3
DC3_IFN
HSPC
ILC
Int.mono
MAIT
NK
NK CD56 Bright
NK_IFN
NKT
Platelet
RBC
T CD4 CM
T CD4 CM_IFN
T CD4 CTL
T CD4 CTL_IFN
T cycling
T CD4 EM
T CD4 EM_IFN
T CD4 Naive
T CD4 Naive_IFN
T CD4 reg
T CD4 reg_IFN
T CD8 CM
T CD8 EM
T CD8 EM_IFN
T CD8 EMRA
T CD8 Naive_IFN
T g/d
pDC

Figure S1. Cell annotation, related to Figure 1.

a) Dot plots displaying gene (left) and protein (right) expression of markers for myeloid and haematopoietic cells. **b)** Dot plots displaying gene (left) and protein (right) expression of markers for B cells. **c)** Dot plots displaying gene (left) and protein (right) expression of markers for T and innate lymphoid cells. **d)** UMAP showing the detailed cell type annotations of B cells, myeloid and progenitors and T cells, respectively. IFN = interferon stimulated, ASDC = Axl Siglec dendritic cell, Dex = dexamethasone, MAIT = mucosal-associated invariant T cell, ASC = antibody-secreting cell, CTL = cytotoxic T lymphocyte, T g/d = gamma delta T cell, EM = effector memory, CM = central memory, EMRA = terminally differentiated effector memory T cell, ILC = innate lymphoid cell, mono = monocyte, int. = intermediate.

Figure S2.

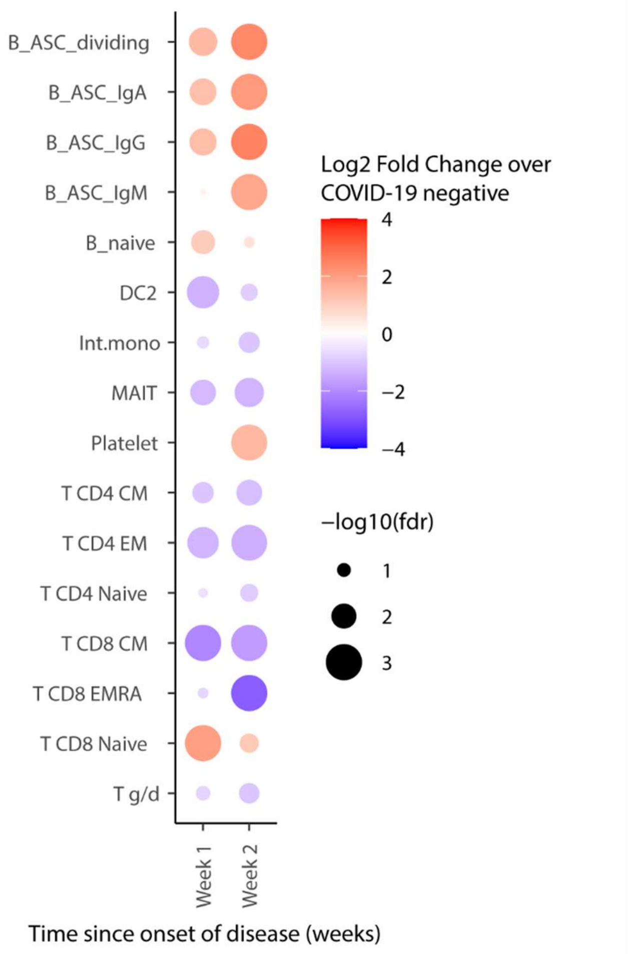

Figure S2. Changes in cell type abundance in COVID-19 over time, related to Figure 2.

Dot plots displaying the significant cell type abundance changes compared to COVID-19 negative samples. Statistical significance of the fold change estimate was measured by the local true sign rate (LTSR), and only abundance changes with BH-adjusted LTSR < 0.05 are shown; values indicate −log10 (FDR). Based on n=37 COVID-19 negative and n=95 COVID-19 positive samples.

Figure S3.

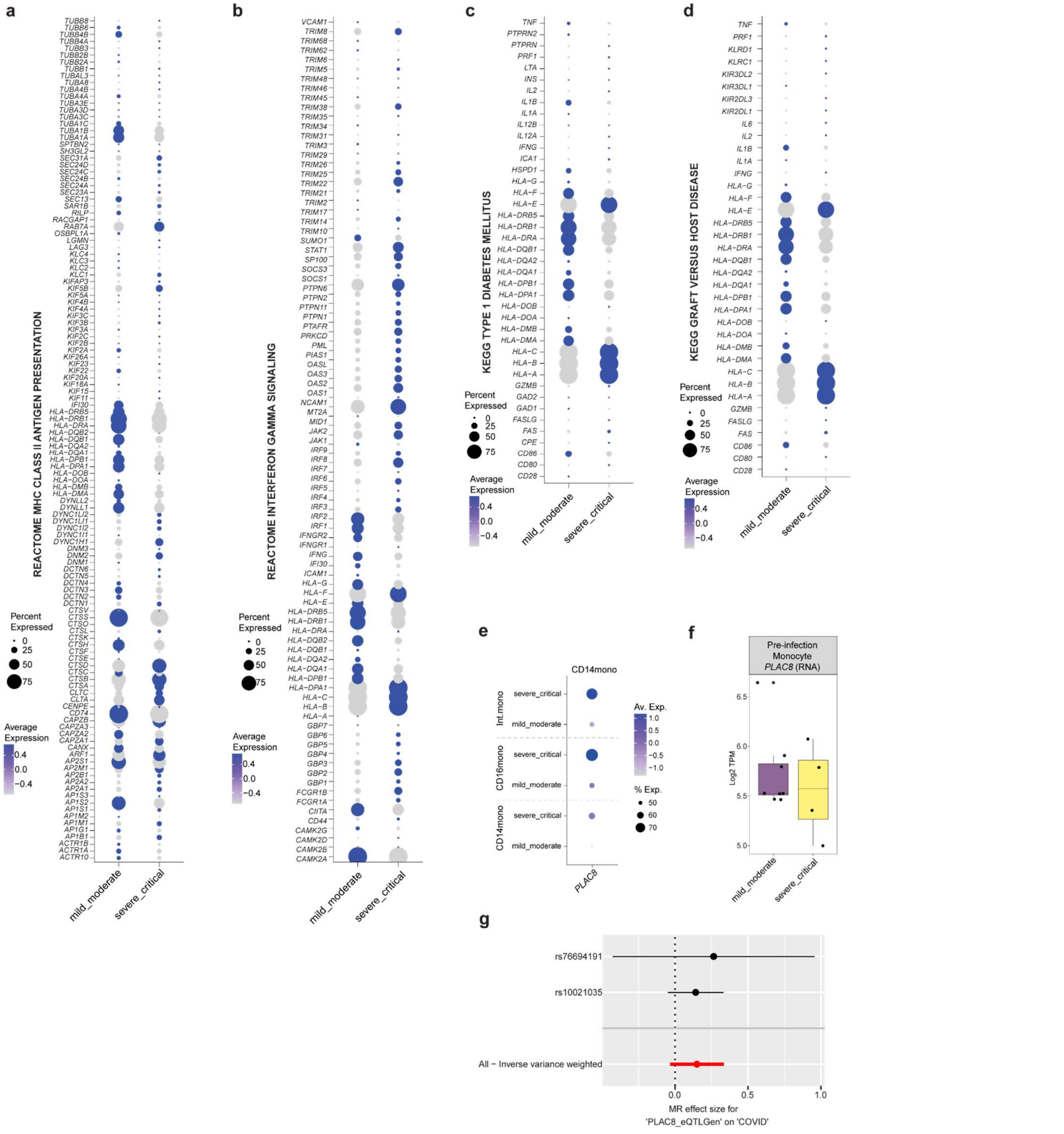

Figure S3. Expression of genes contributing to pathways in CD14 monocytes, related to Figure 3.

Dot plots displaying the expression of genes that contribute to the **a)** Reactome ‘ MHC Class II Antigen Presentation’, **b)** Reactome ‘Interferon Gamma Signalling’, **c)** KEGG ‘ Type 1 Diabetes Mellitus, and **d)** KEGG ‘Graft Versus Host Disease’ pathway terms in CD14 monocytes. **e)** Dot plot displaying the expression of *PLAC8* in monocyte populations, stratified by severity. **f)** Boxplot of *PLAC8* mRNA expression in monocytes (pseudo-bulked data) from pre-infection samples from individuals who were later infected. TPM = transcripts per million. **g)** Forest plot displaying results of a Mendelian randomisation analysis of *PLAC8* gene expression in whole blood on severe COVID-19, showing the MR causal effect estimates and 95% confidence intervals for each instrumental variable and IVW meta-analysis.

Figure S4.

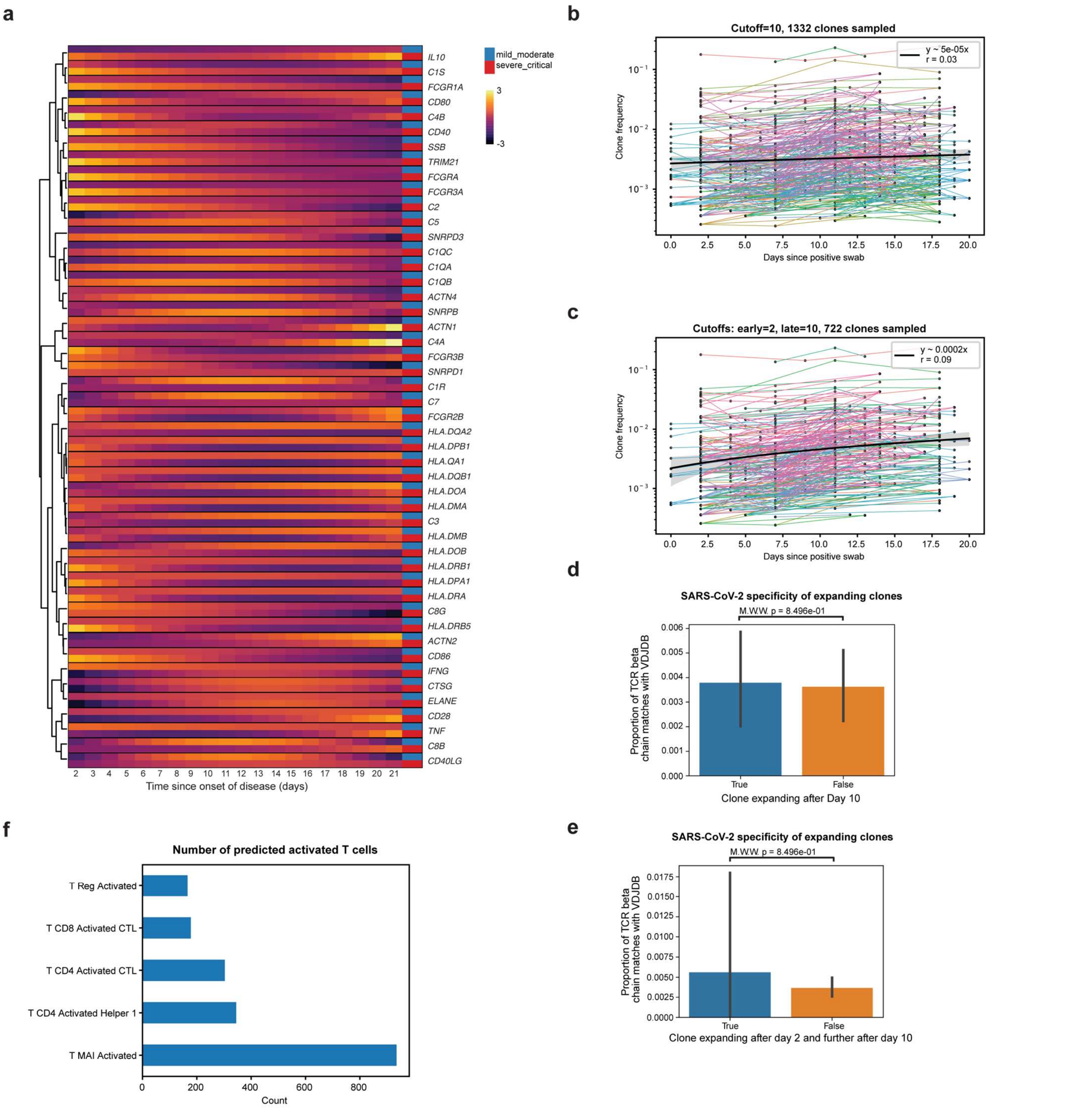

Figure S4. Longitudinal analysis, related to Figure 4.

a) Genes with significantly different temporal dynamics between mild and severe COVID-19 in ESKD patients. Normalized expression for each gene is indicated via the colour gradient, stratified by overall clinical course. **b)** Clonal frequency dynamics for all clones expanded after day 2 post positive PCR result, as well as a trendline. **c)** Clonal frequency dynamics for all clones expanded after day 2 post positive PCR result and further expanded after day 10, as well as a trendline. **d)** Proportion of SARS-CoV-2 specific clones among all clones, stratified by whether the clone expanded after day 10 following positive PCR test. Specificity was determined as a perfect match with a TCR beta chain from the SARS-CoV-2 database VDJDB. M.W.W = two-sided Mann-Whitney-Wilxconon test, p = p-value. **e)** As for d) but stratifying by whether a clone was expanded after day 2 and further after day 10. **f)** Number of activated T cells according to Celltypist predictions, split by T cell type.

Figure S5.

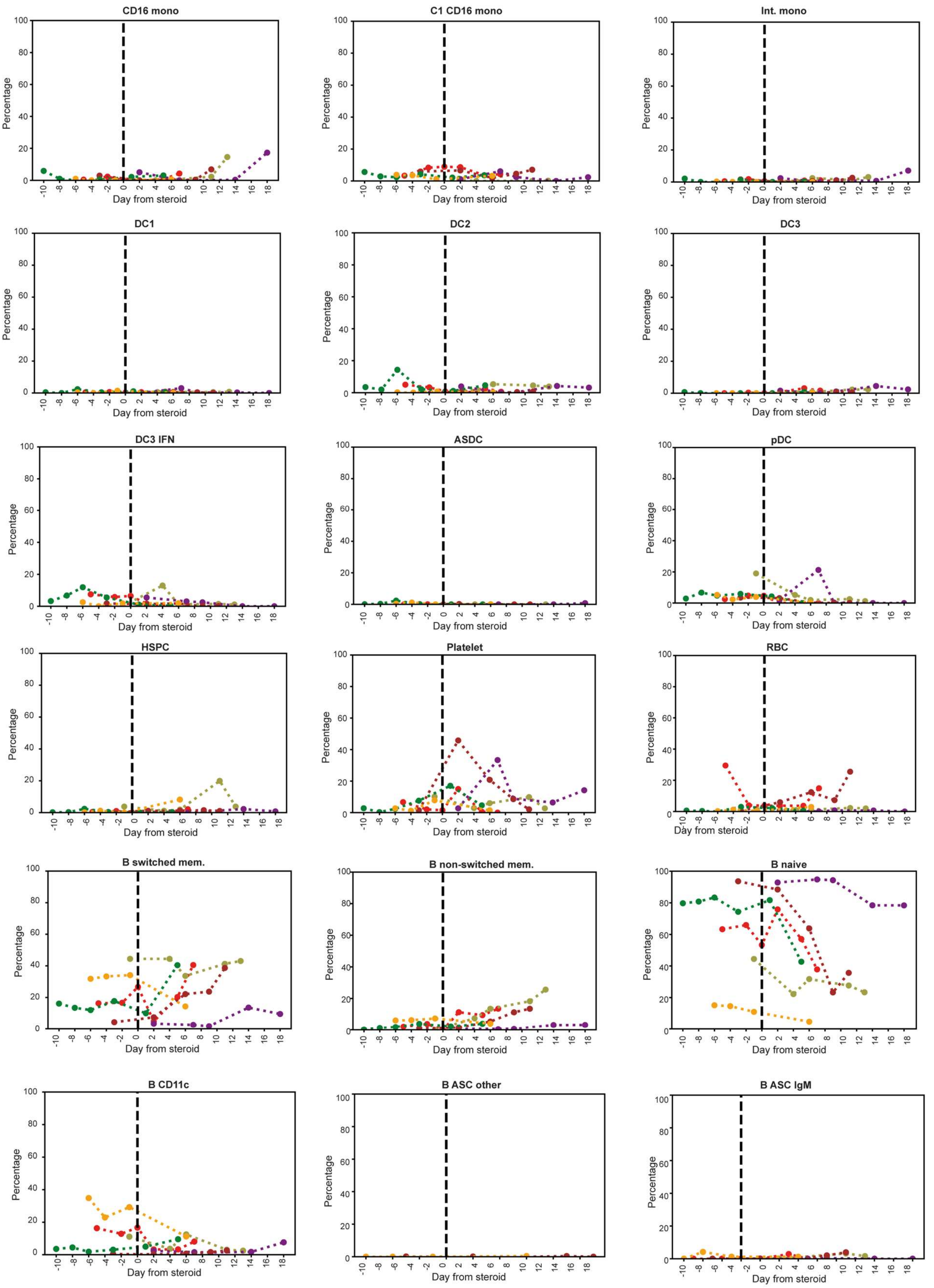

Figure S5. Trend of cell proportions pre- and post- glucocorticoid treatment part 1, related to Figure 5.

Line charts displaying the percentage of cell subsets across the days before and after administration of glucocorticoids. Vertical black dashed line indicates glucocorticoid initiation. Each dot colour and the corresponding dotted lines linking them represent an individual patient.

Figure S6.

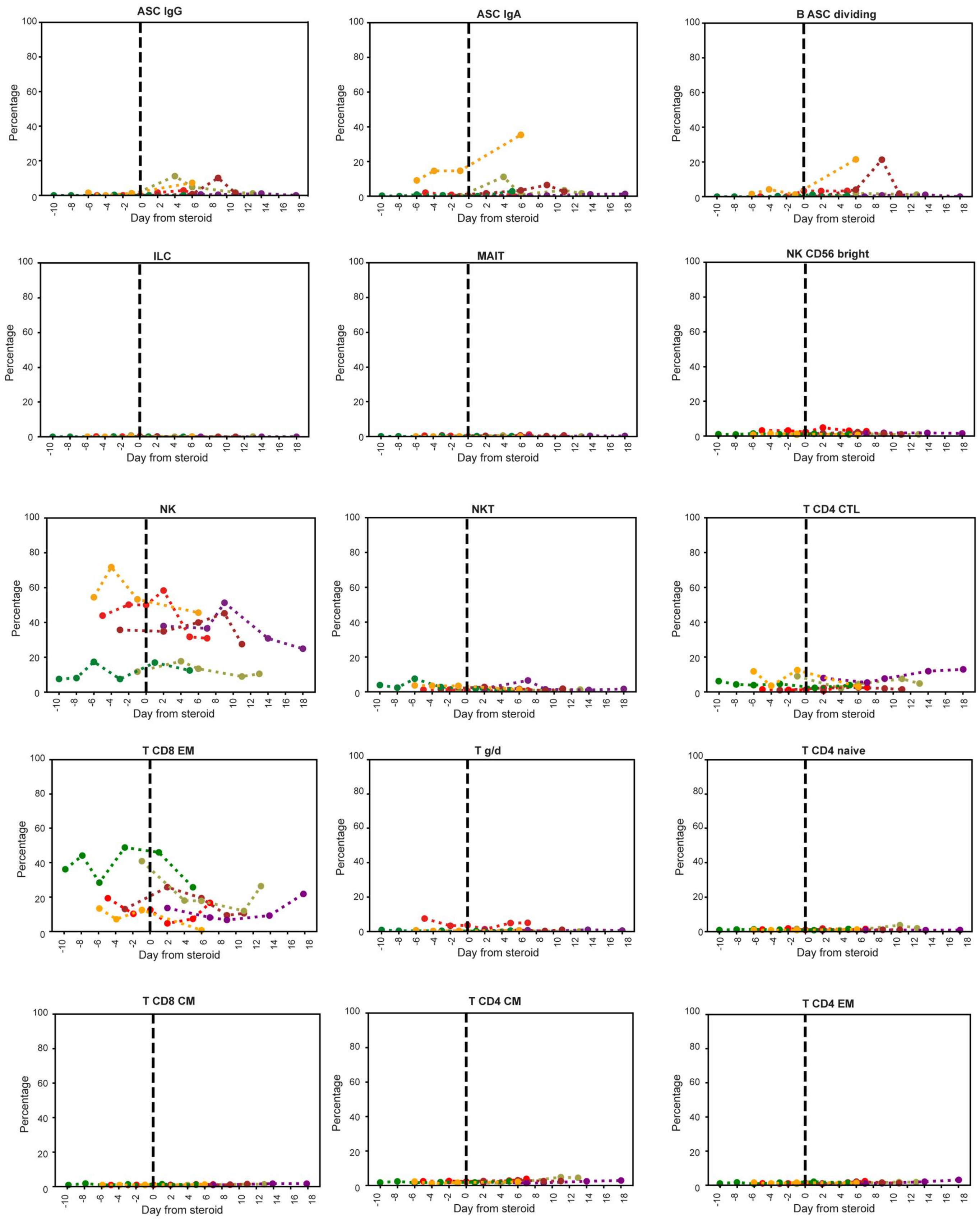

Figure S6. Trend of cell proportions pre- and post- glucocorticoid treatment part 2, related to Figure 5.

Line charts displaying the percentage of cell subsets across the days before and after administration of steroids. Vertical black dashed line indicates glucocorticoid initiation. Each dot colour and the corresponding dotted lines linking them represent an individual patient.

Figure S7.

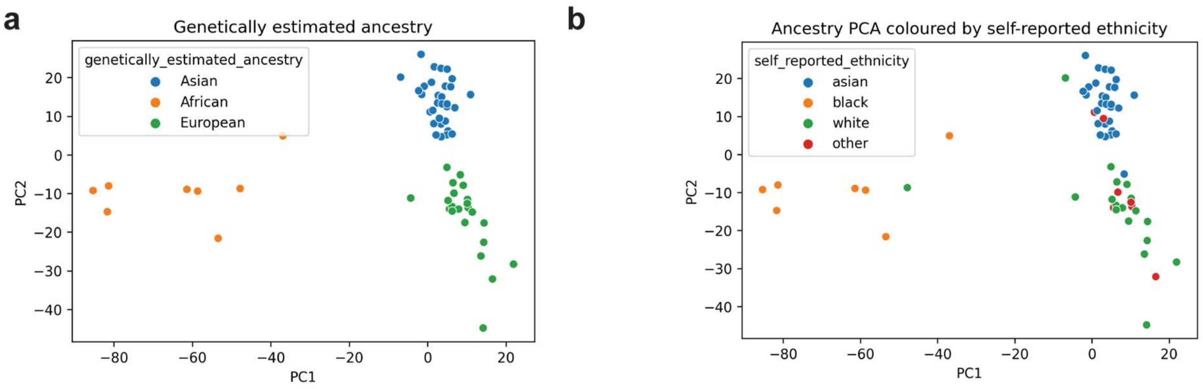

Figure S7. Genetically-inferred ancestry compared to self-reported ethnicity, related to STAR Methods.

Principal components analysis (PCA) plot of the genotype data. PC1= principal component 1, PC2 = principal component 2. Each point represents an individual. Points coloured by **a)** genetically-inferred ancestry, and **b)** self-reported ancestry.
